# Fatty Acid Profile, Oxidative Stability, and Quality Traits of Meat from Broilers Fed Raw or Fermented Rapeseed Cake

**DOI:** 10.3390/foods15111911

**Published:** 2026-05-28

**Authors:** Tatiana Dumitra Panaite, Gabriela Maria Cornescu, Mihaela Dumitru, Florentina Aldea, Ana Elena Cismileanu, Smaranda Mariana Toma, Dan Traian Râmbu, Georgeta Ciurescu, Nicoleta Corina Predescu

**Affiliations:** 1 Laboratory of Animal Nutrition Physiology, National Research-Development Institute for Animal Biology and Nutrition, 1 Calea Bucuresti, 077015 Balotesti, Romania; ana_cismileanu@yahoo.com; 2 Laboratory of Animal Nutrition and Biotechnology, National Research-Development Institute for Animal Biology and Nutrition, 1 Calea Bucuresti, 077015 Balotesti, Romania; mihaela.dumitru@ibna.ro (M.D.); smaranda.pop@ibna.ro (S.M.T.); dan.rambu@ibna.ro (D.T.R.); ciurescu@ibna.ro (G.C.); 3 Institute of Biology Bucharest of Romanian Academy, 296 Splaiul Independentei Street, 060031 Bucharest, Romania; florentina.aldea@ibiol.ro; 4 Faculty of Veterinary Medicine of Bucharest, 105 Splaiul Independentei, District 5, 050097 Bucharest, Romania; corina.predescu@fmvb.usamv.ro

**Keywords:** broiler, raw rapeseed cake, fermented rapeseed cake, meat quality, antioxidant capacity, oxidative stability

## Abstract

Raw rapeseed cake represents a viable alternative protein source for broiler diets, and its fermentation may reduce anti-nutritional factors while improving its feeding value. This 35-day study involved 300 one-day-old ROSS 308 chicks (three groups, four replicates/group, with 25 broilers/replicate) raised on wood shavings (16 broilers/m^2^). Broilers received either a control diet (corn–soybean meal) or diets supplemented with 200 g/kg of RRCs (raw rapeseed cakes) or fermented rapeseed cakes (FRCs). At the end of the trial, eight broilers per group were slaughtered, and breast and thigh samples were collected. The nutritional quality of the meat was assessed by proximate composition, fatty acid profile, and health-related lipid indices. In addition, oxidative status during shelf-life storage was evaluated based on myoglobin content (Mb), metmyoglobin concentration (metMb), total antioxidant capacity (TAC), and lipid peroxidation, determined as thiobarbituric acid reactive substances (TBARS). The omega-3 fatty acid content in fresh breast and thigh samples was significantly higher in RRC and FRC groups compared to C (*p* = 0.014; *p* = 0.0001), which significantly decreased the omega-6/omega-3 ratio for both samples (*p* = 0.0001). The TBARS values exhibited a significant decrease (*p* < 0.0001) between groups for breast and thigh samples. The TAC values showed significantly higher antioxidant capacity in RRC and FRC breasts and thigh samples compared to C, considering both group differences (*p* < 0.0001) and shelf-life evaluation (*p* = 0.001). In thigh samples, the RRC group showed lower metMB values compared to the control group (*p* = 0.042), whereas differences in breast samples were not statistically significant (*p* = 0.054). Healthy lipid indexes registered significantly lower values for experimental groups, both for breast and thigh, and for linoleic/α-linolenic acid ratio (*p* = 0.0001), but for atherogenicity index (AI) (*p* = 0.0001) and thrombogenic index (TI) (*p* = 0.0001) only for the RRC group, while nutritive value index (NVI) significantly increased (*p* = 0.0001) on both groups. In conclusion, RRC and FRC may represent sustainable alternatives to soybean meal in broiler nutrition, supporting improvements in meat lipid profile and oxidative stability. Overall, the RRC group showed more significant effects on n-3 fatty acid enrichment and lipid quality indices, whereas the FRC mainly influenced proximate composition and selected color parameters.

## 1. Introduction

Soybean meal (SBM) remains one of the leading sources of supplementary protein for poultry, renowned for its high quality and consistency [1,2]. Rapeseed meal (RSM), in particular, is a readily available protein source for poultry, but has less protein content compared to SBM [3]. RSM can be used as an economically alternative protein rich in nutrients, with high biological properties, antioxidants, and immunomodulatory capabilities [4,5]. At the same time, its inclusion in diets is restricted by the presence of anti-nutritional factors (ANFs) as presented by Olukomaiya et al. [6]. Rapeseed processing generates several by-products (rapeseed cakes/hulls/oil) with different nutritional characteristics, depending on the extraction and processing methods. Rapeseed cake is the solid by-product remaining after rapeseed oil extraction by mechanical pressing or cold-pressing. The by-product contains a higher residual oil fraction, which increases its energy value while still providing an important source of protein. Depending on the extraction technology, rapeseed processing may produce solvent-extracted meal, expeller-extracted cake, or cold-pressed cake, with important differences in crude fat, crude protein, metabolizable energy, and overall nutritional value [7]. Overall, the agro-food industry by-products, such as rapeseed meal, can be processed efficiently through modern biotechnological methods, which significantly improve their nutritional and energy value as stated by Beszterda and Nogala-Kałucka [8]. Therefore, fermentation using mixed microbial strains is a practical approach to enhance the nutritional value of rapeseed meal. Fermentation, a biotechnological process, facilitates the enzymatic breakdown of ANFs while preserving the integrity of proteins and nutrient absorption [9]. Konkol et al. 2023 [10], confirmed that microbial fermentation using bacteria from the *Bacillus* genus can alter the chemical and biological properties of by-products, including the reduction in ANFs. Furthermore, Bernardeau et al. [11] found that supplementing animal feed with specific *Bacillus* strains can enhance intestinal digestibility, microbiota balance, boost immune function, and improve growth and productive performances. Among the notable species within the *Bacillus* genus with proven positive effects, particularly on young monogastrics with underdeveloped enzymatic systems, include *B. subtilis*, *B. coagulans*, *B. licheniformis*, *B. amyloliquefaciens*, and *B. megaterium* [12]. Therefore, rapeseed meal fermentation presents a viable substitute to soybean meal with significant benefits in enhancing the overall health and poultry meat quality [13,14]. In terms of human health, the fatty acid composition of meat products is an important factor in meat [15]. Including rapeseed in various forms (oil/seeds/meal/cakes) in broilers’ diet can enrich the polyunsaturated fatty acid composition of meat by significantly increasing alpha-linolenic acid [16,17]. Moreover, studies have reported that rapeseed cakes have a positive impact on young animals’ live weight, improving feed efficiency and the European productivity index [18]. Also, the incorporation of enzymatically fermented rapeseed cakes (FRCs) in broiler diets and the addition of phytases, pectinases, and β-glucanase to diets can enhance the coefficient of apparent ileal digestibility and the specific consumption of feed [3]. Previous studies have shown that rapeseed cake and other rapeseed by-products can successfully replace partially soybean meal in broiler diets and may improve the fatty acid composition of meat, particularly through increased deposition of n-3 polyunsaturated fatty acids. Also, rapeseed by-products fermentation is beneficial in reducing ANFs and improving nutrient availability. However, limited information is available on the comparative effects of raw versus fermented rapeseed cake on some broiler meat quality traits and sensory attributes. Therefore, starting from the hypothesis that rapeseed cakes can serve as an alternative to soybean meal—with beneficial effects on broiler meat quality already suggested by previous nutrition studies—this experiment aimed to evaluate whether rapeseed cakes fermentation further improves their value. Specifically, the objective was to investigate and compare the effects of soybean meal-based, raw, and fermented rapeseed cakes dietary inclusion on some broiler meat quality traits.

## 2. Materials and Methods

### 2.1. Acquisition, Fermentation Processing Conditions and Nutritional Assessment of Rapeseed

Firstly, the rapeseed characterized by a glucosinolate content of ≤25 μmol/g and ≤2% erucic acid of the total fatty acids (“double low” variety) was purchased from a local manufacturer from Romania (Expur Group Avril, Ialomita, Romania). To obtain the rapeseed cakes, the seeds were cold-pressed (minimal temperature + 15 °C) using designated pressing oil (Screw Press Farmet Duo 3F, Jiřinková, Czech Republic). For inoculation, *Baccillus subtilis* ATCC 6051 (BS), purchased from the American Tissue Culture Collection (Manassas, VA, USA), was precultured in broth medium (BN, Oxoid, CM0001B g/L: 1 Lab-Lemco’ powder, 2 yeast extract, 5.0 peptone, 5.0 sodium chloride, pH = 7.4 ± 0.2), followed by incubation at 37 °C for 24 h. The selection of *Bacillus subtilis* ATCC 6051a was supported by the safety profile of the species, as it is generally recognized as safe (GRAS) and has been widely used in food- and feed-related fermentation processes. *Bacillus subtilis* ATCC 6051a was selected for fermentation based on its safety profile and enzymatic potential. This strain has been reported to produce amylase, cellulase and protease on different feed raw materials, and recent data on fermented oilseed cakes also indicated protease, cellulase, xylanase and phytase activities. These properties support its potential for improving the nutritional value of pressed oilseed by-products. However, because direct evidence for ATCC 6051a applied specifically to rapeseed cake remains limited, its selection was primarily based on safety and enzymatic bioconversion capacity, while the expected reduction in antinutritional factors was interpreted in relation to previous *Bacillus*-based rapeseed fermentation studies. The strain concentration used in this study was adjusted to 1 × 10^9^ CFU/mL. The solid-state fermentation (SSF) was performed under the optimum conditions as follows: fermentation temperature 37 °C, time 72 h, feed water ratio 1:1 (*w*/*v*), and fermentation moisture content 50% (*w*/*v*) as briefly described by Dumitru et al. [19]. Ultimately, FRC was dried for 2 days, in an oven at 60 °C, and then the dried samples were ground and kept at room temperature until mixed in the broiler diets (20%) for 25 days. Total viable *B. subtilis* counts were determined by serial decimal dilution followed by plating on Nutrient Agar. Appropriate dilutions were plated and incubated at 37 °C for 24 h, after which colonies were counted, and results were expressed as CFU/g. For spore enumeration, aliquots from 10^−1^ were heat-treated at 80 °C for 15 min to inactivate vegetative cells. After cooling, serial decimal dilutions were prepared, plated on Nutrient Agar, and incubated under the same conditions. Spore counts were expressed as CFU/g. Following oven drying, the fermented substrate retained high viable *B*. *subtilis* counts, with 6.15 × 10^10^ CFU/g total viable cells and 1.96 × 10^9^ CFU/g spores. These results confirm that the drying process did not eliminate the viable bacterial population. The raw and fermented rapeseed cake samples were analyzed for nutritional value (Table 1) by an external third-party laboratory to support poultry diet optimization.

The proximate composition was detemined according to the procedures described in Regulation (CE) No. 152/2009 [20], as follows: dry matter content (gravimetric method; SR ISO 6496:2001) [21]; crude protein (Kjeldahl method; SR EN ISO 5983-2:2009) [22]; crude fat (Soxhlet extraction; SR ISO 6492:2001) [23]; crude fiber (intermediate filtration method; SR EN ISO 6865:2002) [24]; ash content (gravimetric method; SR EN ISO 2171:2010) [25]. Total starch was determined using the Total Starch HK (Hexokinase) Assay Kit. From the analytical data, OM (organic matter), and NFE (nitrogen-free extractives) were calculated as follows: NFE (%) = OM (%) − [CP (%) + EE (%) + CF (%)]; OM (%) = DM (%) − Ash (%). The amino acid profile was analyzed by acid hydrolysis using ion chromatography with UV detection (IC-UV) (ISO 13903:2005) [26]. The glucosinolates were determined by spectrophotometry in raw and fermented rapeseed cake samples, according to the Hungarian Feed Codex 2004, Chapter III, 61 [27]. The phytic acid was determined using an enzymatic–spectrophotometric method, based on the measurement of phosphorus released by phytase and alkaline phosphatase. Phenolic content was determined using the classic Folin–Ciocâlteu method; antioxidant activity was analyzed using the DPPH (2,2-diphenyl-1-picrylhydrazyl) method; flavonoid concentration was analyzed using a spectrophotometric method based on the interaction of flavonoid compounds with aluminum ions. A gravimetric tannin determination technique based on binding with insoluble polyvinylpyrrolidone (PVP) was assessed. The in vitro nutrients digestibility (dDM, dOM, and dCP) was determined using 2-steps successive incubations with pepsin and pancreatin enzymes.

### 2.2. Experimental Design (Animals, Housing and Diets)

The experiment complied with Directive 2010/63/EU on the protection of animals used for scientific purposes and the experimental procedures. The feeding trial was conducted in the experimental halls of the National Research-Development Institute of Animal Biology and Nutrition (IBNA-Balotesti, Romania) according to the experimental protocol (no. 1200/13.03.2024) approved by the Commission of Ethics of the institute. A 35-day feeding trial was conducted on 300 one-day-old broiler chicks Ross 308 obtained from a local hatchery. Before being purchased from the farm, all birds were vaccinated against Marek’s disease and bronchitis at the hatchery. The broilers were housed in an experimental hall and kept on permanent wood shavings litter, with controlled environmental conditions. The light regimen was adequate for the birds’ age (23 h light/1 h darkness), and they had free access to feed and water. The broilers were randomly assigned to 3 experimental groups. Each group had 4 replicate pens (25 broilers per pen; 100 broilers/group). For meat quality analysis, 2 birds per pen from each group (8 birds/group) were randomly selected for slaughtering to collect the meat samples. For the first 10 days (starter phase), all broilers were fed the same basal diet. During the grower and finisher phases, birds were allocated to different dietary treatments based on feed formulation: a soybean meal–corn-based diet for control (SBM), a diet containing raw rapeseed cake (RRC), and another diet containing fermented rapeseed cake (FRC). In the RRC and FRC groups, rapeseed cakes were included at 200 g/kg of diet (Table 2).

The diets (in mash form) were optimized by Brill^®^ formulation software (version 2.6; Cargill Feed Management Systems/Format Solutions, Florham Park, NJ, USA), using the chemical analysis of each ingredient and according to the Ross Broiler Management Handbook [28]. After the manufacture of the compound feed, the bags were properly labeled for each experimental batch and stored in a room with controlled temperature (22 ± 2.15 °C) throughout the experimental period.

At the end of the feeding trial (35 days), a total of 24 broilers (8 broilers/group; 2 birds/pen/group) were randomly selected and slaughtered to collect the meat samples (thigh and breast) to assess their nutritional quality. A total of 192 meat samples (breast and thigh) were collected per experiment, immediately after slaughtering. Each chicken breast or thigh sample was cut into 4 portions and randomly divided into 4 analysis sections. For each section was used a total of 48 meat samples (24 thigh and 24 breast samples): 48 fresh meat samples were used to determine proximate composition, collagen, pH value and fatty acids analysis (section one); 48 fresh meat samples for drip loss evaluation (section two); 48 frozen meat samples were analyzed for texture parameters (third section); 48 frozen meat samples were analyzed for TBARS, TAC, metmyoglobin, myoglobin analysis, color and sensory evaluation (forth section). Frozen meat samples were vacuum-packed and frozen at −22 °C. At the time of analysis, the meat samples were removed from the freezer and left to thaw slowly in the refrigerator (0–4 °C), kept in the vacuum packaging during thawing to prevent moisture loss through evaporation and external contamination. When the temperature in the thermal center of the sample, measured internally with a digital thermometer, reached + 2 °C, the sample was processed for the respective analysis (The sensory evaluation study was approved by the IBNA Scientific Research Ethics Committee (no. 124/18.12.2023), with the participation of personnel from USAMV Bucharest, and it was carried out in the Biochemistry Laboratory of the Faculty of Veterinary Medicine in Bucharest, under controlled temperature conditions (22 ± 0.5 °C) and incandescent lighting of approximately 500 lx.

### 2.3. Chemical and Physical Properties of Meat Samples

#### 2.3.1. Chemical Properties of Meat Samples

##### Proximate Composition and pH Measurements of Meat Samples

For chemical composition assessment, approximately 300 g/meat samples, sliced and homogenized, were used to determine the moisture, protein, fats, and collagen using a near-infrared reflectance (NIR) analyzer, PerkinElmer DA6200 (PerkinElmer Inc., Waltham, MA, USA), according to a method previously described by Ciurescu et al. [29]. Meat sample pH measurements were assessed at 24 h post mortem using a portable pH meter (Five Go F2-Food kit with LE 427IP67, Sensor Mettler-Toledo GmbH, Greifensee, Switzerland).

##### Fatty Acid Profile and Health Lipid Indices of Meat Samples

Fatty acids (FAs) were determined by Perkin Elmer-Clarus 500 gas chromatograph (PerkinElmer, Inc., Shelton, CT, USA) with flame ionization detector (FID) fitted with a BPX70 capillary column (60 m × 0.25 mm i.d., 0.25 μm film thickness) using methyl ester fatty acid (FAME) according to with ISO/TS 17764–2 (2008), as described by Turcu et al. [30]. FAME identification was performed by comparing the retention times of the standards, and the results were expressed as g of fatty acid per 100 g of total fatty acids, but the results were reported as mg FA/100 g meat samples. Based on the results obtained for fatty acids, the meat quality indices were calculated and classified according to the qualitative indices (SFA/UFA, saturated fatty acid/unsaturated fatty acid; PUFA/MUFA, polyunsaturated fatty acid/monounsaturated fatty acid; PUFA/SFA, polyunsaturated fatty acid/saturated fatty acid; n-6/n-3 ratio, omega 6 fatty acids/omega 3 fatty acids ratio [31]; LA/ALA, linoleic acid/alpha-linolenic acid ratio [32]; EPA + DHA, sum of eicosapentaenoic acid and docosahexaenoic acids [33,34]; UI, unsaturation index [35], and nutritional attributes (PI, peroxidability indices [36]; NVI, nutrition value index [37]; IA, index of atherogenicity [38]; IT, index of thrombogenicity [38]; HH ratio, hypocholesterolemic/hypercholesterolemic [39]; and HPI, health-promoting index [40]).

##### Myoglobin Concentration and Metmyoglobin Percentage

Meat homogenate was prepared 0, 7, and 14 d of storage by homogenizing the ground meat with 10 volumes of 40 mM sodium phosphate buffer (pH 6.8) using a Glas-Col Variable Speed Reversible Homogenizer (Glas-Col LLC, Terre Haute, IN, USA) for 30 s at maximum speed. An ice bath was used to prevent the increasing of temperature and protein denaturation during extraction. The homogenate was centrifuged at 8000× *g* for 15 min at 4 °C. After centrifugation, the supernatant was divided into two tubes (for myoglobin/metmyoglobin and TAC determination). The supernatant was read at 525, 545, 565, and 572 nm. The contents of myoglobin and metmyoglobin were calculated on the basis of the following Equations (1) and (2) and expressed as milligrams of myoglobin per gram of meat:

Myoglobin concentration (mg/g meat) = (−0.166 R1 + 0.086 R2 + 0.088 R3 + 0.099) × A_525_ × 0.0175 × dilution factor (1)

Metmyoglobin content (%) = (−2.514 R1 + 0.777 R2 + 0.800 R3 + 1.098) × 100 (2)

where A_525_ is absorbance at 525 nm, R1 is A_572_/A_525_, R2 is A_565_/A_525_, and R3 is A_545_/A_525_.

##### Antioxidant Activity of Meat Samples

The antioxidant activity of the homogenized meat samples was assessed by measuring their scavenging abilities to DPPH stable radicals. A sample of 100 μL was mixed with 0.004 mM DPPH solution prepared in 95% MeOH to a final volume of 4 mL and allowed for reaction development in the dark, for 1 h at room temperature. The absorbance of the resulting solution and the blank were recorded spectrophotometrically at 517 nm. The inhibition of free radicals by DPPH in percent (%) was calculated by using the following Formula (3):

Inhibition (%) = [(A blank − A sample)/A blank] × 100(3)

##### Lipid Peroxidation of Meat Samples

Malondialdehyde (MDA) serves as a reliable indicator for assessing lipid peroxidation in meat samples. It reacts with thiobarbituric acid to form a red adduct with an absorption peak at 532 nm. TCA-TBA-HCl reagent contains 15% (*w*/*v*) trichloroacetic acid, 0.375% (*w*/*v*) thiobarbituric acid, and 0.25 N hydrochloric acid. Mix 0.5 g of the biological sample until disintegrated with 2.0 mL of the TCA-TBA-HCl reagent. After centrifugation at 5000× *g* for 15 min at 4 °C, the supernatants were heated in a boiling water bath for 15 min. Allow the solution to cool and measure the absorbance of the supernatant at 532 nm against a blank containing all reagents except the lipid. The concentration of MDA is calculated using an extinction coefficient of 1.56 × 10^5^ M^−1^ cm^−1^, and the results were expressed as mg MDA/kg.

#### 2.3.2. Physical Properties of Meat Samples

##### Texture Profile Analysis of Meat Samples

The meat texture profile for breast and thigh samples was determined at room temperature (≈20 °C) from fresh samples (*n* = 8) using a Brookfield CT3 Texture Analyzer (Version 3.0; AMETEK Brookfield, Middleboro, MA, USA) fitted with a 50 kg load cell. Cylindrical cores (20 mm diameter, 15 mm height) were extracted from the thigh and breast muscles using a handheld stainless steel cork borer, sampled parallel to the longitudinal orientation of the muscle fibers. Each sample was placed in the middle of a rectangular fixture base table and compressed perpendicular to the fiber direction using a TA11 clear acrylic cylindrical probe (25.4 mm diameter) (AMETEK Brookfield, Middleboro, MA, USA). Following the methodology described by Ciurescu et al. [29] with some modification, a double compression cycle was performed to 50% of the sample height at a constant speed of 1.5 mm s^−1^ (pre-test), 2.0 mm s^−1^ (test), and 2.0 mm s^−1^ (post-test), with a trigger force of 20 g used to initiate the measurement. Finally, TexturePro CT V1.6 software was used to determine hardness, gumminess, chewiness, springiness, adhesiveness, resilience, and cohesiveness, based on four readings.

##### Instrumental Color of Meat Samples

Meat color was measured using a portable colorimeter 3 nh YS3020 (Shenzhen Threenh Technology Co., Ltd., Beijing, China), according to the CIE L*a*b* color system established by the Commission Internationale de l’Éclairage [41]. The color parameters recorded were L* (lightness), a* (redness), and b* (yellowness). Measurements were performed using the D65 illuminant, an 8 mm aperture, and a 10° standard observer angle according to the method described by Panaite et al. [42] and Mancini et al. (2020) [43]. Before each measurement session, the instrument was calibrated using the supplied white calibration tile. For each meat sample, three consecutive readings were taken at different points on the exposed muscle surface, avoiding visible connective tissue, fat deposits, and surface defects. The mean of the three readings was used as the individual sample value for further statistical analysis.

##### Meat Sample Drip Loss Measurements

Drip loss was measured as an indicator of water-holding capacity using the suspension (bag) method. At 24 h post-mortem, a number of 8 meat samples/group (≈30–50 g) were collected from the breast (*Pectoralis major*) and thigh muscles, cut according to muscle fiber orientation (horizontal or vertical relative to the circular knife), and manually trimmed to remove skin, visible fat, and connective tissue. The samples were then cut into uniform portions (10, 20, and 30 mm in diameter), gently blotted with absorbent paper to remove surface moisture, and weighed to record the initial weight (W_0_). Each portion was placed in an airtight plastic bag and suspended to prevent contact with the bag surface, then stored at 4 °C for 24 and 48 h. After storage, samples were removed, gently blotted again, and reweighed to obtain the final weight (W_1_). Drip loss was calculated as

Drip loss (%) = [(W_0_ − W_1_)/W_0_] × 100. (4)

##### Meat Sample Sensory Evaluation

Sensory analysis was performed to evaluate the organoleptic attributes of chicken meat samples at different storage days (0, 7, 14 days) based on an intensity scoring system used to assess odor (fresh meat) and texture (firmness, elasticity, consistency). A total of 48 meat (breast and thigh sample pieces ≈ 50–100 g/sample) were stored under controlled refrigerated conditions (0–4 °C) using vacuum packaging and/or modified atmosphere packaging, according to the experimental design. Prior to evaluation, samples were randomly numbered, removed from refrigeration, and brought to room temperature 30 min before testing. Sensory evaluation was conducted in a well-ventilated room under neutral lighting conditions (white light), under room temperature conditions (20–22 °C), following the general recommendations of ISO 8589:2007; Sensory Analysis—General Guidance for the Design of Test Rooms [44]. Before the sensory evaluation, written informed consent was obtained from all panelists. The voluntary participants, semi-trained assessors (10), were informed about the purpose and nature of the test, the samples to be consumed and evaluated, any potential risks, their right to withdraw at any time, and data confidentiality. Prior to the evaluation, they received instructions regarding the evaluation procedure, scoring system, and descriptors used in order to ensure consistency and repeatability of the assessments. During evaluation, each panelist received individual assessment forms and coded samples, and each sample was analyzed separately in the predetermined order. They were instructed not to consume strongly flavored foods or beverages, tobacco, or chewing gum for at least 1 h before the test. These instructions were provided to ensure a consistent understanding of the evaluation criteria and to improve the reliability and reproducibility of the sensory assessments. Between samples, panelists cleansed their palate with still water to reduce carryover effects. Each sensory attribute was rated using a 0–5 intensity scale, where 0 indicated the absence of the attribute, and 5 indicated a very high intensity score. The evaluated attributes included odor and texture intensity, which were scored as follows: 0 = absent or not perceived, 1 = very weak, 2 = weak, 3 = moderate, 4 = intense, and 5 = very intense.

### 2.4. Statistical Analysis

One-way analysis of variance (ANOVA), using StatView for WINDOWS (SAS, version 6.0 SAS Institute Inc., Cary, NC, USA), was carried out to determine the effects of treatments on meat quality parameters (proximal analysis, pH, fatty acids profile, healthy fatty indexes, meat textural properties), according to the following linear model:

Y_ij_ = μ + T_i_ + e_ij_

where Y_ij_ is the mean of the jth observation of the ith treatment; μ is the sample mean; T_i_ is the effect of the ith treatment; and e_ij_ is the effect of the error. To evaluate the normality and homogeneity of experimental data, Shapiro–Wilk’s test and Levene’s test were applied.

For parameters evaluated during storage period (0, 7, and 14 days), including color-related biochemical markers, instrumental meat color traits, and sensory attributes of chicken meat, data obtained were analyzed using repeated measures ANOVA design where “sample” is nested within “treatment”, General Linear Model (GLM) procedures of SAS (Statistical Analysis System, Minitab version 17, SAS Institute Inc., Cary, NC, USA), followed by Tukey’s test, using the following statistical model:

Y_ijk_ = µ + α_i_ + β_j_ + α_i_β_j_ + e_ijk_

where Y_ijk_ = variable measured for the kth observation of the ith treatment and jth period; μ is the sample mean, α_i_ is the effect of the ith treatment; βj is the effect of the jth period; αiβj = interaction of ith treatment and jth period, and e_ijk_ is the effect of error. Different letters (uppercase or lowercase) indicate statistically significant differences between groups (*p* < 0.05).

The graphs for oxidative stability (TAC and TBARS) and drip loss were statistically analyzed using GraphPad Prism 10.2.0 software (GraphPad Software, La Jolla, CA, USA), with different letters indicating significant differences between groups (*p* < 0.05).

## 3. Results

### 3.1. Nutritional Value Assessment of Raw and Fermented Rapeseed Cakes

The fermentation process of rapeseed cakes (Table 1) improved their nutritional composition. Crude protein content increased by 2.35 percentage points, while crude protein digestibility increased by 4.14 percentage points. In contrast, crude fiber content decreased by 0.57 percentage points. Fermentation also enhanced the antioxidant-related profile, as reflected by increases in polyphenols (+1.63 units), antioxidant capacity (+21.12 units), and flavonoids (+0.13 units). At the same time, the concentrations of antinutritional factors were reduced, including glucosinolates (−1.80 µmol/g), phytic acid (−0.56 percentage points), and tannins (−2.18 mg/g), whereas free phosphorus increased by 0.09 units, representing more than a twofold increase. Based on the fatty acid profile information provided in Table 1, the fatty acid composition of the two ingredients was generally similar, with the exception of alpha-linolenic acid (ALA, 18:3n-3) and total omega-3 fatty acids, which showed a lower numerical concentration in the FRC compared to the RRC.

### 3.2. Proximate Composition and Meat Quality Traits of Broilers Fed Raw and Fermented Rapeseed Diets

Our results concerning proximate composition, collagen and pH value of thigh and breast chicken meat samples are presented in Table 3. Following meat quality evaluation, moisture content was significantly lower within the FRC group for breast (*p* = 0.0001) samples compared with the SBM and RRC groups, whereas in thigh meat samples, the decrease was significant (*p* = 0.007) only compared to the RRC group. Conversely, collagen content was significantly higher in the FRC group in both breast (*p* = 0.0001) and thigh (*p* = 0.001) samples when compared to the SBM and RRC groups. Meat pH values were not affected by diet, compared to values across groups in both breast and thigh samples.

### 3.3. Fatty Acid Composition of Thigh and Breast Meat Samples from Broilers Fed Raw and Fermented Rapeseed Diets

The effects of raw and fermented rapeseed diets on the fatty acid composition of broiler meat are presented in Table 4 for both thigh and breast samples.

In thigh samples, total saturated fatty acids (ΣSFA) were significantly higher in the SBM group than in the RRC and FRC groups (*p* = 0.001). Among individual SFAs, the capric acid (C10:0) differed significantly among groups (*p* = 0.043), with higher values in SBM and FRC than in RRC. For the myristic acid (C14:0), a significant difference was observed (*p* = 0.035), with SBM showing higher values than FRC, while no difference was detected between SBM and RRC. Palmitic acid (C16:0) exhibited a highly significant diet effect (*p* = 0.0001), with the highest levels in SBM compared with both RRC and FRC. Within the monounsaturated fatty acid fraction (ΣMUFA), several individual MUFAs differed significantly among groups. Myristoleic acid (C14:1) and palmitoleic acid (C16:1) were significantly higher in the SBM group compared to both RRC and FRC groups (*p* = 0.0001 for each). Similarly, erucic acid (C22:1n-9) was significantly higher (*p* = 0.009) in SBM compared with RRC and FRC groups, and nervonic acid (C24:1n-9) showed the same pattern (*p* = 0.0001), indicating an overall enrichment of these MUFAs in the SBM treatment compared to the two experimental groups. Within the ΣPUFA fraction, the only n−6 PUFA showing a diet effect was dihomo-γ-linolenic acid (C20:3n−6), which was significantly higher (*p* = 0.004) in the SBM group compared to the RRC and FRC groups. Regarding total n−3 PUFA (Σn−3), the RRC group exhibited the highest values (*p* = 0.0001) compared to the SBM and FRC groups. Within Σn−3, α-linolenic acid (C18:3n−3) was significantly higher in RRC and FRC compared to SBM (*p* = 0.0001). In contrast, eicosatrienoic acid (C20:3n−3) differed significantly among groups (*p* = 0.003), with higher values in the SBM compared to the FRC group. Consistent with these changes, the n−6/n−3 ratio was significantly lower in RRC and FRC compared with SBM (*p* = 0.0001). The SFA/UFA ratio was highest in SBM relative to RRC and FRC (*p* = 0.0001), whereas the PUFA/SFA ratio was highest in RRC compared with SBM and FRC (*p* = 0.0001). In breast samples, total saturated fatty acids (ΣSFA) were significantly higher (*p* = 0.0001) in the SBM and FRC groups compared to the RRC group. Among individual SFAs, the C14:0 registered a highly significant statistical difference (*p* = 0.0001) in the SBM and FRC groups compared to the RRC group. The pentadecanoic acid (C 15:0) registered the highest statistical value (*p* = 0.0001) in the FRC group compared to the SBM and RRC groups. The fatty acids C16:0 (*p* = 0.0001) and octadecanoic acid (C 18:0) (*p* = 0.005) recorded statistically significant values within the SBM and FRC groups compared with the RRC group. The eicosanoic acid (C 20:0) registered the highest values (*p* = 0.011) for the SBM group compared to the FRC group. Among the monounsaturated fatty acid fraction (ΣMUFA) determined within breast samples, C 14:1 and C 16:1concentrations in the SBM group registered statistically significant values (*p* = 0.0001) compared to RRC and FRC groups. The pentadecenoic acid (C 15:1) registered significant statistical values (*p* = 0.002) in the SBM and RRC groups compared to the FRC group. The cis-oleic acid (C 18:1n9c) registered the highest values (*p* = 0.006) on FRC and RRC groups compared to the SBM group. On the other hand, significantly higher values (*p* = 0.0001) were observed for the nervonic acid (C24:1 n-9) on the SBM and RRC groups compared to the FRC group. In the breast muscle, the diet significantly affected the polyunsaturated fatty acid profile. Total PUFA (ΣPUFA) was statistically higher (*p* = 0.037) in the SBM group compared to the FRC group. A similar pattern was observed for total n-6 PUFA (Σn-6), which presented higher values (*p* = 0.007) in the SBM group compared to the FRC group. The C18:2n-6 acid followed the same trend, being higher in the SBM group (*p* = 0.017) compared to the FRC group. In contrast, γ-linolenic acid (C18:3n-6) increased significantly (*p* = 0.0001) in the FRC group compared to the SBM and RRC groups. For arachidonic acid (C20:4n-6), the SBM and RRC groups showed comparable values, and both presented higher values (*p* = 0.006). compared the FRC group. Also, the dietary treatments significantly influenced the n-3 fatty-acid fraction and several lipid quality indices. Total n-3 PUFA and C18:3n-3 were significantly influenced by diet, with the RRC group showing a more favorable n-3 profile compared to the SBM and FRC groups (*p* = 0.014). Higher significant values (*p* = 0.009) were observed in the C20:3n-3 concentration of the SBM group compared to the FRC group. In parallel, the n-6/n-3 ratio differed significantly among groups (*p* = 0.0001), with the best ratio values observed in the RRC and FRC groups compared to the SBM group. Significant changes were registered in SFA/UFA (*p* = 0.0001), where the best values were observed for RRC groups compared to SMB and FRC groups. As for PUFA-related indices, the highest ration PUFA/MUFA (*p* = 0.005) was registered in the SBM group compared to the FRC group, and the highest significant value (*p* = 0.001) for PUFA/SFA ratio was noticed in the RRC and SBM groups compared to the FRC group.

### 3.4. Health-Related Lipid Indices of Thigh and Breast Meat Samples from Broilers Fed Raw and Fermented Rapeseed Diets

Table 5 summarizes the main health-related lipid indices of thigh and breast meat, highlighting the results obtained following the dietary inclusion of raw and fermented rapeseed.

For thigh samples, the quality indices, the LA/ALA ratio, differed significantly among groups. The SBM group showed a significantly higher value (*p* = 0.0001) compared to both the RRC and FRC groups. Significant differences were observed for all nutritional indices calculated (*p* < 0.05). The nutritional value index (NVI) was significantly higher (*p* < 0.001) in RRC and FRC groups compared to SBM. The atherogenic index (AI) showed significant differences (*p* < 0.001), with the highest value in the SBM group, compared to the RRC group. A similar pattern was found for the thrombogenic index (TI), where SBM had the highest value (*p* < 0.001) compared to FRC and RRC groups. The hypocholesterolemic/hypercholesterolemic (HH) ratio also differed significantly (*p* < 0.001), with the highest value in the RRC group, compared to the SBM group. For the health-promoting index (HPI), SBM showed higher values (*p* = 0.026) compared to the RRC group; the metabolic indices were significantly influenced by diet (*p* < 0.05), and elongase activity was significantly higher (*p* = 0.001) in the RRC and FRC groups compared to the SBM group. The Δ9-desaturase activity differed significantly among groups (*p* = 0.015), with FRC showing higher values compared to SBM. Consistently, Δ9-desaturase (C16:1 + C18:1) was higher (*p* < 0.001) in the RRC and FRC groups compared to the SBM group. The combined Δ5 + Δ6 desaturase index also differed statistically (*p* = 0.024), with higher values in the RRC group compared to the FRC group. The activity index was significantly higher in the RRC and FRC groups compared to SBM (*p* < 0.001). For the breast muscle, the quality indices calculated, the LA/ALA ratio, were significantly different between all groups (*p* < 0.001). The SBM group registered the highest value, compared to the RRC group. The NVI was higher (*p* < 0.001) in the RRC and FRC groups compared to the SBM group. For AI, RRC registered a lower significant value (*p* < 0.001) compared to SBM and FRC groups. The same pattern was observed for TI (*p* < 0.001), where RRC was lower than SBM and FRC. The HH ratio differed significantly (*p* < 0.001), being highest in the RRC group compared to the SBM and FRC groups. HPI also differed statistically (*p* = 0.003), with RRC showing the lowest value, compared to SBM and FRC groups. The metabolic indices, elongase activity differed between all groups (*p* < 0.001), with the highest significant value registered on the RRC group compared to the SBM group. Thioesterase activity was significantly lower (*p* < 0.001) in the FRC group compared to the SBM and RRC groups. For Δ9-desaturase, FRC had higher values (*p* = 0.015) compared to the SBM group. Similarly, Δ9-desaturase estimated from (C16:1 + C18:1) registered higher values in RRC and FRC groups compared to SBM (*p* < 0.001). The Δ5 + Δ6 desaturase index statistically differed (*p* = 0.034), being higher in the RRC group compared to the FRC group. The activity index presented higher values (*p* = 0.005) in the RRC group compared to the values observed in the FRC group.

### 3.5. Meat Texture Profile Parameters of Broilers Fed Raw and Fermented Rapeseed Diets

The effects of raw and fermented rapeseed diets on meat texture parameters of broiler meat quality are presented in Table 6.

In thigh meat, texture profile analysis indicated no significant differences (*p* > 0.05) among dietary groups for hardness, adhesiveness, resilience, cohesiveness, springiness, gumminess, or chewiness. Overall, these results suggest that the experimental diets did not register significant differences in the texture parameters of thigh muscle samples. In breast meat, the texture profile analysis showed a significant effect (*p* = 0.0001) of diet only on the cohesiveness parameter. The FRC group showed a significantly lower value (*p* = 0.0001) for the cohesiveness parameter compared with the SBM and RRC groups. No significant differences (*p* > 0.05) were noticed between dietary groups for hardness, adhesiveness, resilience, springiness, gumminess, or chewiness.

### 3.6. Oxidative Stability Parameters of Broiler Meat Samples as a Result of Raw and Fermented Rapeseed Cake Dietary Inclusion

#### 3.6.1. Myoglobin Concentration and Metmyoglobin Percentage Assessed in Broiler Meat

Table 7 presents the results of the myoglobin concentration and metmyoglobin percentage determined in broiler meat samples in response to the dietary inclusion of raw and fermented rapeseed cakes.

#### 3.6.2. Antioxidant Activity and Lipid Peroxidation Assessed in Broiler Meat

**Figure 1:**
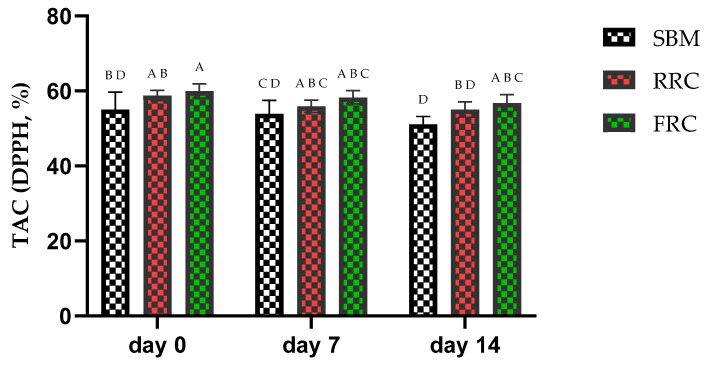
Effects of dietary inclusion of raw and fermented rapeseed cakes on total antioxidant capacity (TAC; DPPH, %) of thigh meat during storage (0, 7, and 14 days); different uppercase letters (A–D) above columns indicate statistically significant differences (*p* ≤ 0.05). SBM, soybean meal control diet; RRC, raw rapeseed cake diet; FRC, fermented rapeseed cake diet.

**Figure 2:**
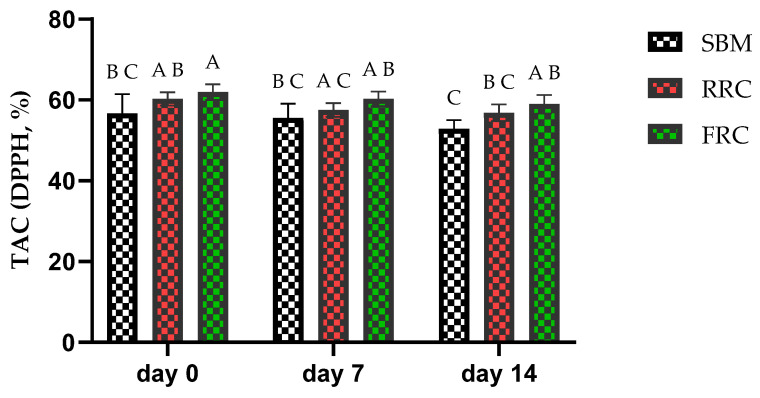
Effects of dietary inclusion of raw and fermented rapeseed cakes on total antioxidant capacity (TAC; DPPH, %) of breast meat during storage (0, 7, and 14 days): different uppercase letters (A–C) above columns indicate statistically significant differences (*p* ≤ 0.05). SBM, soybean meal control diet; RRC, raw rapeseed cake diet; FRC, fermented rapeseed cake diet.

**Figure 3:**
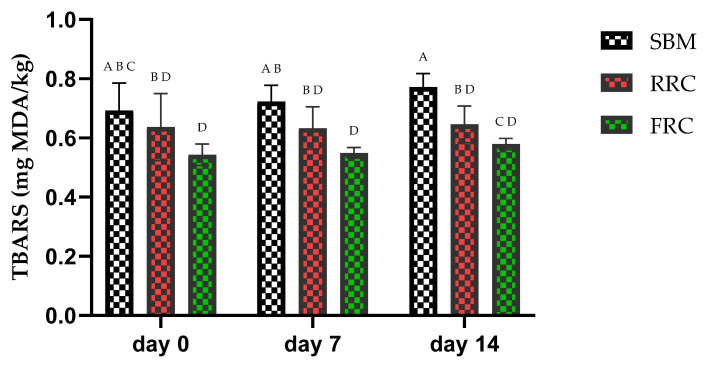
Effects of dietary inclusion of raw and fermented rapeseed cakes on thiobarbituric acid reactive substances (TBARS; mg MDA/kg) in thigh meat samples during storage (0, 7, and 14 days); different uppercase letters (A–D) above columns indicate statistically significant differences (*p* ≤ 0.05). SBM, soybean meal control diet; RRC, raw rapeseed cake diet; FRC, fermented rapeseed cake diet.

**Figure 4:**
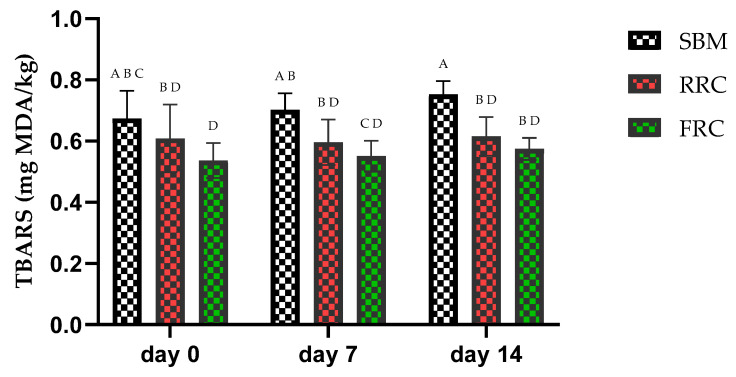
Effects of dietary inclusion of raw and fermented rapeseed cakes on thiobarbituric acid reactive substances (TBARS; mg MDA/kg) in breast meat samples during storage (0, 7, and 14 days). different uppercase letters (A–D) above columns indicate statistically significant differences (*p* ≤ 0.05). SBM, soybean meal control diet; RRC, raw rapeseed cake diet; FRC, fermented rapeseed cake diet.

The results of the antioxidant activity and lipid peroxidation determined in broiler meat samples in response to the dietary inclusion of raw and fermented rapeseed cakes are presented in Figure 1, Figure 2, Figure 3 and Figure 4.

In thigh meat samples, storage period significantly influenced pigment and antioxidant status. Myoglobin content (Table 7) decreased with storage period length (*p* = 0.002), and total antioxidant capacity (Figure 1) also declined significantly over time (*p* = 0.001), indicating a progressive reduction in color-related pigment stability and antioxidant potential during refrigerated storage. Regarding diets, significant differences (*p* = 0.042) were detected for metmyoglobin (metMB), the SBM group showing the highest pigment oxidation compared to experimental groups. Diet registered a significant effect on oxidative status, as TAC (*p* < 0.0001) was higher in the RRC and FRC groups compared to the SBM group, while TBARS values (*p* < 0.0001) (Figure 3) were reduced by rapeseed supplementation, reflecting improved lipid oxidative stability. No period × group interactions were observed (*p* > 0.05).

In breast meat, the storage period significantly affected both pigment oxidation and antioxidant status. As presented in Table 7, myoglobin concentration decreased as the storage period increased (*p* = 0.009), while metmyoglobin increased (*p* = 0.006), demonstrating a possible pigment oxidation. TAC values (Figure 2) decreased concomitantly with storage period (*p* = 0.001), consistent with a gradual decreasing of antioxidant defenses during storage period. Dietary treatment significantly influenced TAC (*p* < 0.0001), with the experimental groups showing higher antioxidant capacity compared to the SBM group, and also significantly (*p* < 0.0001) influenced the TBARS concentration (Figure 4), with supplementation reducing lipid oxidation relative to the control. There was no significant period × group interaction noticed (*p* > 0.05).

### 3.7. Color Parameters of Broiler Meat Samples During Different Storage Periods as a Result of Dietary Inclusion of Raw and Fermented Rapeseed Cakes

Table 8 presents the effects of raw and fermented rapeseed diets on color chicken meat parameters during different storage periods (0, 7, 14 days). In thigh meat, the storage period did not significantly influence lightness (L*), redness (a*), or yellowness (b*) parameters (*p* > 0.05). In contrast, dietary group significantly influenced all three color coordinates: L* (*p* = 0.041), a* (*p* = 0.039), and b* (*p* = 0.001).

Overall, the FRC group showed lower values for L* and b* parameters compared to the SBM and RRC groups. A statistically significant (*p* = 0.019) period × group interaction was detected for a* parameter, indicating that the dietary differences in redness varied across storage time. In breast meat, the period effect was significant only for the b* parameter (*p* = 0.0001), showing a storage-related significant change in yellowness, while L* and a* parameters were not influenced by storage period (*p* > 0.05). The group effect was significant for L* (*p* = 0.0001) and a* (*p* = 0.0001) parameters, indicating that dietary treatment changed breast lightness and redness/greenness, whereas the b* parameter did not differ among groups (*p* > 0.05). In addition, a statistically significant (*p* = 0.002) period × group interaction was observed for the b* parameter, suggesting that the evolution of breast yellowness during storage depended on the diet.

### 3.8. Drip Loss of Broiler Meat During Different Storage Periods as a Result of Raw and Fermented Rapeseed Dietary Inclusion

**Figure 5:**
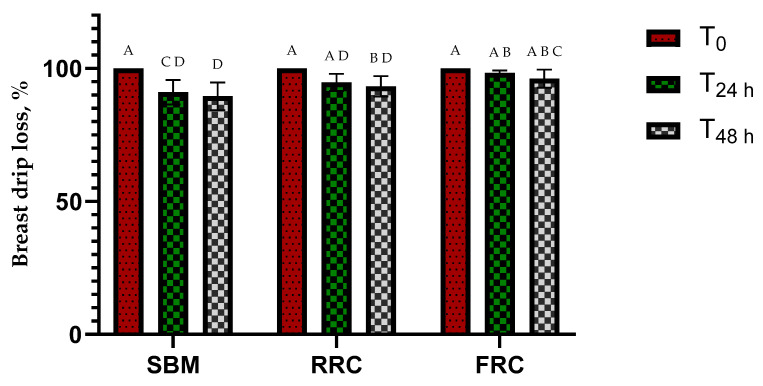
Effect of dietary inclusion of raw and fermented rapeseed cakes on drip loss (%) in breast meat during storage (0, 24, and 48 h); different uppercase letters (A–D) above columns indicate statistically significant differences (*p* ≤ 0.05). SBM, soybean meal control diet; RRC, raw rapeseed cake diet; FRC, fermented rapeseed cake diet; T0, initial time; T24 h, after 24 h; T48 h, after 48 h.

**Figure 6:**
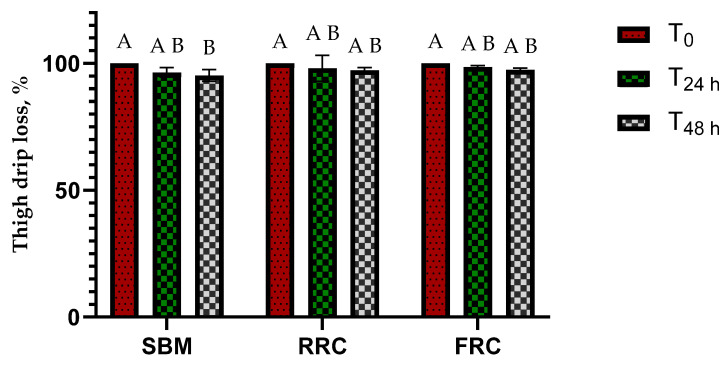
Effect of dietary inclusion of raw and fermented rapeseed cakes on drip loss (%) in thigh meat during storage (0, 24, and 48 h): different uppercase letters (A,B) above columns indicate statistically significant differences (*p* ≤ 0.05). SBM, soybean meal control diet; RRC, raw rapeseed cake diet; FRC, fermented rapeseed cake diet; T0, initial time; T24 h, after 24 h; T48 h, after 48 h.

The effects of dietary inclusion of raw and fermented rapeseed cakes on the drip loss parameter in samples of breast (Figure 5) and thigh meat (Figure 6) were assessed at various storage times (0, 24, and 48 h). For thigh samples, after 24 h of storage, the highest water retention values were registered in the FRC and RRC groups compared to the SBM group. This pattern was maintained after 48 h, when both rapeseed-fed groups continued to register higher water retention compared to the SBM group. Overall, the results indicate a gradual reduction in water retention over time, with a significantly higher drip loss percentage (*p* < 0.05) in the SBM group, compared with the RRC and FRC groups, suggesting lower water-holding capacity. A similar time-dependent pattern was recorded for breast samples. At 24 and 48 h, FRC and RRC groups registered the highest water retention capacity, compared to SBM groups, which indicates that storage time negatively affected breast meat water retention in all groups, although this decrease was less observed in the rapeseed-fed groups, especially FRC.

### 3.9. Meat Sensory Evaluation Across Different Storage Periods in Broilers Fed Raw and Fermented Rapeseed Diets

The effects of raw and fermented rapeseed diets on meat sensory evaluation during different storage periods (0, 7, and 14 days) are presented in Table 9. For the storage period main effect, all attributes (odor intensity, firmness, consistency, and elasticity) were significantly affected in both meat samples (*p* = 0.0001).

In the thigh samples, odor intensity and firmness decreased significantly (*p* = 0.0001) gradually from day 0 to day 14. Similarly, consistency and elasticity declined statistically (*p* = 0.0001) gradually from day 0 to day 14. A similar pattern was observed in the breast samples, with all evaluated meat attributes showing highly significant (*p* = 0.0001) changes across the different storage periods. Regarding the group main effect, significant differences were observed mainly for odor intensity and firmness, whereas consistency and elasticity (*p* = 0.552) were not significantly influenced by group. In the thigh samples, odor intensity was higher in the RRC and FRC groups compared to the SBM group (*p* = 0.0001). Firmness was also affected by group (*p* = 0.001), with FRC values higher compared to the SBM group, while group effects were not significant for consistency (*p* = 0.063) or elasticity (*p* = 0.552). In the breast meat samples, odor intensity differed significantly among groups (*p* = 0.0001), with RRC and FRC values higher compared to SBM. Firmness was significantly influenced by group (*p* = 0.0001), with higher values in the FRC group compared to the SBM group; group effects were not significant for consistency (*p* = 0.220) or elasticity (*p* = 0.294). For the period × group interaction, a significant interaction was detected for the thigh meat samples in odor intensity (*p* = 0.008) and firmness (*p* = 0.012), indicating that differences among groups depended on storage time. In contrast, interactions were not significant for thigh consistency (*p* = 0.370) and elasticity (*p* = 0.833). In the breast meat samples, no significant period × group interactions were found for any attribute (odor *p* = 0.068; firmness *p* = 0.129; consistency *p* = 0.363; elasticity *p* = 0.747), suggesting that group-related differences were generally consistent across storage periods.

## 4. Discussion

### 4.1. Effects of Fermentation Process on Nutritional Value of Raw Rapeseed Cakes

Fermentation significantly improved the nutritional value of raw rapeseed cake, obtaining a higher crude protein content and digestibility, together with lower crude fiber, glucosinolate, phytic acid, and tannin concentrations. The increase in free phosphorus polyphenols, flavonoids, and antioxidant capacity indicates that fermentation enhanced not only the nutritive value but also the functional bioactivity of rapeseed cakes.

The nutritional changes observed in our study about the fermented rapeseed cake can be explained by the specific activity of *Bacillus subtilis* ATCC 6051a during solid-state fermentation. During fermentation, B. subtilis ATCC 6051a can use part of the available carbohydrates and soluble nutrients for growth and sporulation, while simultaneously secreting extracellular enzymes that modify the substrate matrix. This strain has been reported to produce amylase, cellulase and protease on different feed raw materials, and recent data obtained on oilseed cakes fermented with B. subtilis ATCC 6051a showed protease, cellulase, xylanase and phytase activity, supporting its capacity to improve the nutritional value of pressed oilseed by-products [19]. The higher numerical values of CP and dCP of the FRC group (values reported are means of three determinations/batches/products) can be due to the growth of *B. subtilis* ATCC 6051a microbial biomass protein contribution to the fermented rapeseed cake. At the same time, proteolytic enzymes secreted during fermentation can hydrolyze rapeseed storage proteins into smaller peptides and free amino acids, which may improve protein solubility and digestibility, as reported by Zhu et al. [45]. Our results are similar to the higher total phenolics, flavonoids, and DPPH scavenging activity post-fermentation observed by Wang et al. [46], who showed that fermented rapeseed meal contained more soluble peptides and free amino acids and exhibited stronger antioxidant activity and higher biological availability. The literature reported that fermentation improvement level depends on several factors: raw ingredient form (meal or cake), residual oil level, microbial strain, enzyme supplementation, moisture, temperature, and fermentation duration [45].

### 4.2. Effects of Raw and Fermented Rapeseed Diets on Broilers’ Meat Proximate Composition and Meat Quality

Our results regarding meat sample chemical composition showed that FRC dietary inclusion altered the water content of the thigh and breast muscle, mainly by lowering moisture and increasing collagen, without any significant changes in pH values. Çapan & Bağdatli [47], in a study investigating chicken thigh and breast meat produced by organic and conventional methods, reported normal values for moisture in breast, 73.76–74.21%, and in thigh, 74.39–74.79%. The moisture content of raw chicken breast generally ranges around 73–75%, while raw thigh meat usually ranges around 74.4–76.6% [48]. The lower moisture content observed in both thigh and breast samples from the FRC group may indicate a reduced water-holding capacity, potentially associated with the higher collagen proportion, but these compositional changes were not accompanied by significant differences in hardness, chewiness, gumminess, or springiness. This may indicate that, at the inclusion level used, the observed collagen differences were not sufficient to produce significant texture improvements of broiler meat. On the other hand, other studies suggested that the combination of lower moisture and higher collagen could determine a firmer muscle structure, as reported by Mir et al. [49], while rapeseed meal, including fermented forms, may also affect technological meat properties related to water retention [10]. However, other studies replacing SBM with RSM in broilers’ diets have reported minimal changes concerning meat-quality traits, highlighting that responses depend on inclusion level, diet formulation, and birds’ genotype [50]. In our study, meat pH was not influenced by diet, suggesting a similar postmortem glycolytic pattern across all groups. In contrast, previous studies indicate that replacing soybean meal with rapeseed ingredients may not have a significant effect on most meat-quality traits overall, although changes in muscle pH have been reported in some cases. These discrepancies likely reflect differences among studies in birds’ genotype, the type and processing of the rapeseed ingredient, and the postmortem time chosen for pH measurement [51,52].

### 4.3. Effects of Raw and Fermented Rapeseed Diets on Fatty Acid Composition of Thigh and Breast Samples

In our study, the analyzed thigh muscle samples presented a lower ΣSFA concentration in the RRC and FRC groups compared to the SBM group, together with the lower SFA/UFA ratio and the lower n-6/n-3 ratio, likely attributable to the overall dietary fatty acid composition of the diets, rather than solely to the inclusion of rapeseed, whether raw or fermented, as a single ingredient. Although fermentation was supposed to improve the nutritional characteristics of raw rapeseed, no improvement was noticed regarding changes in fatty acid profiles between RRC and FRC analyzed. Meat samples: Similar results with our study were reported by Gao et al. 2020 [16]. Also, the lower erucic acid values observed in RRC and in FRC in thigh samples compared with SBM may be due to the dietary lipid profile of the diet or fatty acid metabolism in different types of meat. A significant reduction in C14:0 and C16:0 is important, as these fatty acids contribute to the saturated fraction and are considered less desirable for human nutrition than monounsaturated and n-3 polyunsaturated fatty acids [16]. The same author found that both raw and fermented rapeseed cake improved several lipid quality traits, but not always to the same extent, while Drażbo et al. [53] reported that raw rapeseed cake produced the highest n-3 PUFA concentrations and the most desirable n-6/n-3 ratio in turkey breast muscle. In the breast muscle, the RRC group produced the lowest ΣSFA and the most favorable SFA/UFA ratio compared to the FRC and SBM groups. At the same time, SBM showed higher ΣPUFA and Σn-6 values compared to the FRC group, mainly due to higher linoleic acid (C18:2n-6) and arachidonic acid (C20:4n-6). These results are consistent with studies in broilers showing that lowering the dietary n-6: n-3 ratio increases tissue n-3 deposition and improves the nutritional value of meat, even when total PUFA does not increase in parallel [54]. Our study also noticed an increase in α-linolenic acid (C18:3n-3) in both experimental groups, especially in the breast and thigh muscles, compared to the SBM group. Rapeseed-feed ingredients are known as effective sources for enriching poultry meat with n-3 fatty acids [55] in a short period of time, and the n-6/n-3 lowering ratio observed in RRC and FRC groups is also consistent with the capacity of rapeseed to affect a lipid profile considered more beneficial for consumers. Abdulla et al. [56] showed that changing the dietary n-6:n-3 ratio affected the expression of genes involved in lipid metabolism, suggesting that the type of dietary fat can influence both fatty acid deposition and metabolic activity in poultry tissues. In our study, a stronger observable dietary effect in the thigh muscle than in the breast muscle is expected, because the thigh muscle contains more oxidative fibers and more intramuscular fat, whereas the breast muscle is predominantly glycolytic. According to Liu et al. [57], these may explain why the beneficial changes in ΣSFA, Σn-3, the n-6/n-3 ratio, and the PUFA/SFA ratio were particularly evident in the thigh samples.

### 4.4. Effects of Raw and Fermented Rapeseed Diets on Health Indices

The significantly lower LA/ALA ratio observed in RRC and FRC groups, in both thigh and breast muscles, combined with a higher NVI value, indicates a more favorable balance between n-6 and n-3 precursors compared to the SBM group. Similar results were reported by Kralik et al. [58], who showed that rapeseed oil lowered the LA/ALA ratio in broiler meat compared with sunflower oil dietary inclusion. Likewise, Semwogerere et al. [59] registered a lower n-6: n-3 ratio in the meat of hens fed expeller-pressed canola meal compared to birds on a conventional soybean-based diet. In breast samples, the RRC group showed lower AI and TI values and higher HH compared to the SBM group. For the metabolic indices, in the thigh, both RRC and FRC had higher elongase activity, higher Δ9-desaturase (C16:1 + C18:1), and a higher activity index compared to the SBM group, while the FRC group also had higher Δ9-desaturase compared to the SBM group. In the breast, the most evident favorable changes were seen in RRC, especially for elongase activity, while both RRC and FRC increased Δ9-desaturase (C16:1 + C18:1) compared to the SBM group. Similar findings were reported by Gao et al. [16], who showed that fermented rapeseed cake improved the fatty acid profile of breast meat and reduced the breast n-6: n-3 ratio at 28 days, and by Ashayerizadeh et al. [60], who found that fermented rapeseed meal decreased SFA and increased PUFA in thigh meat, while also reducing thigh cholesterol and malondialdehyde levels. These health indices evaluated in animal-origin food are valuable comparative tools designed for assessing the nutritional quality of FA profiles in meat samples, rather than predicting specific health outcomes in individuals. In the present study, the n-6/n-3 ratio was reduced in the RRC and FRC groups compared with SBM, particularly in thigh meat, where values decreased from 46.94 in SBM to 27.64 in RRC and 32.25 in FRC. However, although these reductions indicate statistically favorable changes compared to a soybean meal-based diet, all values remained substantially higher than the commonly recommended dietary range of approximately 4:1–5:1. Therefore, in this context, this improvement should be regarded as a relative enhancement of meat lipid quality.

### 4.5. Effects of Raw and Fermented Rapeseed Diets on Meat Texture Parameters

Our study shows that partial replacement of soybean meal with RRC or FRC did not significantly affect thigh meat texture, as hardness, adhesiveness, resilience, cohesiveness, springiness, gumminess, and chewiness parameters registered similar values among groups. These results suggest that rapeseed-based diets did not significantly affect the main factors of texture in thigh muscle. In breast meat, diet affected only cohesiveness, which was lower in the FRC group compared to the SBM and RRC groups. This may indicate a slight change in muscle structure or water retention, without a general deterioration of texture, since the other texture parameters were not significantly influenced [61].

### 4.6. Effects of Raw and Fermented Rapeseed Diets on Meat Oxidative Stability Parameters

The experimental results show that refrigerated storage progressively affected pigment stability and antioxidant protection in broiler meat, as reflected by myoglobin and TAC decreasing in both thigh and breast samples and by the concomitant increase in metmyoglobin in breast meat samples. This pattern is consistent with the normal postmortem changes occurring in poultry meat during chilled storage, when myoglobin is progressively oxidized to metmyoglobin and antioxidant defenses decline. As a result, discoloration and oxidative deterioration increase over storage time [62]. The lower metmyoglobin values in thigh meat and the higher TAC, together with reduced TBARS in both thigh and breast samples from RRC and FRC-fed broilers, suggest that rapeseed inclusion improved meat oxidative stability compared with the SBM diet, due to rapeseed bioactive compounds, which exert antioxidant activity. In addition, fermentation can reduce glucosinolates and other antinutritional factors while improving nutrient availability, which may further support antioxidant status in broilers. Similar findings were reported by Ashayerizadeh et al. [60], who showed that fermented rapeseed meal reduced malondialdehyde levels in thigh muscle and increased TAC in both breast and thigh muscles, in agreement with existing evidence that dietary antioxidants deposited in tissues can enhance meat quality and prolong shelf-life. Gołębiewska et al. [63] reported that, in broilers fed diets containing oxidized rapeseed oil supplemented with vitamin E and polyphenols, the antioxidant status of the tissues was significantly improved.

### 4.7. Effects of Raw and Fermented Rapeseed Diets on Meat Shelf-Life Color Parameters

In broiler meat, color parameters depend mainly on the concentration and chemical state of heme pigments, especially myoglobin, but also on muscle pH, protein denaturation, water distribution, and oxidation processes. Both storage conditions and diet composition may alter the L*, a*, and b* color coordinates [49]. Dietary treatment significantly affected all thigh color coordinates, with the FRC group showing lower L* and b* values compared to the SBM and RRC groups. This indicates that fermented rapeseed cake modified the meat color, making it darker and less yellow. Konkol et al. [10] observed that fermented rapeseed meal altered color traits, particularly b*, while Ashayerizadeh et al. [60] also reported that fermented rapeseed meal affected meat quality traits in broilers. Gao et al. (2020) [16] showed that fermented rapeseed cake modifies tissue fatty acid composition, which may indirectly influence color through altered oxidative susceptibility of muscle lipids and pigments. In breast meat, storage period influenced only the b* parameter, whereas L* and a* parameters remained unaffected. This selective sensitivity of yellowness is in agreement with previous results on refrigerated chicken breast, where storage time affected b* without changes in L* or a* parameters. Kaewthong et al. (2019) [64] found that chilled chicken breast maintained relatively stable L* and a* values, while b* changed over time.

### 4.8. Effects of Raw and Fermented Rapeseed Diets on Drip Loss During Different Storage Periods

Our study’s results showed a progressive increase in drip loss during refrigerated storage observed in both thigh and breast meat samples, which corresponds to the normal postmortem evolution of water distribution in poultry meat. During cold storage, muscle proteins change, and the fibers contract, so the meat retains less water. As a result, more fluid gradually accumulates over time [65]. In our study, a high-water retention capacity for thigh and breast meat samples was observed, especially in the FRC group, but also in the RRC, compared to the SBM group. Our findings are in agreement with previous reports showing that broilers fed rapeseed meal exhibited higher water-holding capacity in thigh muscle, indicating reduced water loss [50]. Moreover, earlier evidence suggests that differences in water-holding capacity become more pronounced as postmortem storage progresses [66]. In line with this, Bowker and Zhuang [67] reported that breast filets with lower water-holding capacity accumulate greater drip loss during storage, attributing this effect to postmortem protein alterations that diminish the muscle’s ability to retain water.

### 4.9. Effects of Raw and Fermented Rapeseed Diets on Meat Sensory Evaluation Parameters

The decline in firmness, consistency, and elasticity observed during storage is due to the postmortem storage, which is accompanied by proteolytic changes, moisture redistribution, exudate formation, and gradual weakening of the myofibrillar structure, all of which can soften the meat matrix. In chicken breast, Sujiwo et al. [68] found that shear force decreased significantly during storage, supporting the interpretation that chilled storage reduces structural resistance. The finding that RRC and especially FRC improved odor intensity and firmness relative to SBM is biologically plausible in the context of rapeseed fermentation. Fermentation can reduce antinutritional compounds such as glucosinolates and phytate, while improving nutrient availability and amino acid [69]. Drażbo et al. [53] showed that fermentation considerably reduced glucosinolates and phytate-phosphorus in rapeseed cake, while Wu et al. [69] demonstrated improved ileal digestibility of several amino acids in fermented rapeseed meal.

## 5. Conclusions

In Romania, rapeseed cultivation has increased in recent years, with official data from the Romanian Ministry of Agriculture and Rural Development indicating approximately 702,000 ha cultivated in 2025. This increasing production may improve the availability of rapeseed by-products, which can be valorized as a local alternative protein source in broiler diets. From a practical perspective, farmers and feed manufacturers may be interested in partially replacing soybean meal with rapeseed cake, particularly when this ingredient is locally available and nutritionally suitable. Fermentation could further increase its practical value by reducing anti-nutritional factors and improving nutrient utilization. In our study, the inclusion of raw and fermented rapeseed cakes in broilers’ diets influenced some meat quality traits compared with the soybean meal control group. Fermented rapeseed cakes influenced proximate composition by reducing moisture and increasing collagen content, especially in breast meat, without affecting the pH value. Both rapeseed-based diets improved the fatty acid profile by reducing SFA and a more favorable ratio between n-6 and n-3 fatty acids, with these effects being more evident in thigh meat. Among the two treatments, raw rapeseed cake generally produced the most favorable responses in terms of Σn-3, PUFA/SFA ratio, and health-related lipid indices, while fermented rapeseed cake retained some benefits, particularly regarding the n-6/n-3 balance. Although refrigerated storage progressively reduced oxidative stability, both rapeseed-based diets improved the oxidative status of the meat compared with soybean meal, as reflected by higher total antioxidant capacity, lower lipid oxidation, and reduced metmyoglobin formation in thigh meat. Growth performance, carcass yield, organ development, and economic efficiency data were not included in the present manuscript, limiting the ability to fully evaluate the practical replacement of soybean meal with rapeseed cake; this is the subject of another manuscript. Only one inclusion level of rapeseed cakes, raw and fermented, was tested, preventing dose–response interpretation. In addition, the relatively small number of samples per group, based on eight samples per meat type/group, could represent a limitation and may have influenced the statistical power. Also, the meat samples analyzed at 0 days that were subjected to a freeze–thaw cycle prior to measurement at all three time periods (0, 7, and 14 days), which may have influenced the oxidative and color parameters. Overall, these findings suggest that raw rapeseed cake was more effective in improving the fatty acid profile of the meat, whereas fermented rapeseed cake had a greater influence on compositional and color traits. Both feeding solutions may therefore be considered valuable alternatives to soybean meal in broiler nutrition, contributing to improved nutritional quality and oxidative stability of the meat.

## Figures and Tables

**Table 1:** Chemical composition, antioxidant profile, anti-nutritional factors and fatty acid profile/classification of raw and fermented rapeseed cakes.

Specification	RRC	FRC
Proximate composition (%)		
DM	91.42 ± 0.27	90.10 ± 0.36
dDM	54.52 ± 0.16	52.51 ± 0.21
OM	85.86 ± 0.25	84.37 ± 0.34
dOM	54.76 ± 0.16	53.43 ± 0.21
CP	30.44 ± 0.10	32.79 ± 0.12
dCP	68.65 ± 0.21	72.79 ± 0.28
EE	14.51 ± 0.03	9.85 ± 0.06
CF	10.79 ± 0.03	10.22 ± 0.04
Ash	5.56 ± 0.02	5.73 ± 0.02
NFE	29.26 ± 0.10	31.51 ± 0.11
Starch	0.07 ± 0.001	0.16 ± 0.001
Amino acids, g/100 g		
Ornithine	<0.01	<0.01
Serine	1.32 ± 0.01	1.33 ± 0.00
Methionine	0.62 ± 0.03	0.61 ± 0.00
Phenylalanine	1.22 ± 0.01	1.17 ± 0.00
Leucine	2.09 ± 0.01	2.09 ± 0.01
Lysine	1.56 ± 0.01	2.03 ± 0.01
Hydroxyproline	0.30 ± 0.00	0.32 ± 0.001
Aspartic acid	2.14 ± 0.01	2.15 ± 0.01
Cysteine + Cystine	0.82 ± 0.00	0.80 ± 0.00
Glycine	1.58 ± 0.01	1.56 ± 0.01
Glutamic acid	5.32 ± 0.03	5.49 ± 0.02
Arginine	1.64 ± 0.01	1.84 ± 0.01
Proline	2.07 ± 0.01	1.88 ± 0.01
Alanine	1.4 ± 0.01	1.33 ± 0.01
Threonine	1.39 ± 0.01	1.34 ± 0.01
Valine	1.57 ± 0.01	1.49 ± 0.01
Histidine	0.75 ± 0.00	0.83 ± 0.00
Antioxidant profile		
Polyphenols (mg/g GAE)	8.54 ± 0.04	10.17 ± 0.05
Antioxidant capacity (µM Trolox/g)	54.57 ± 0.28	75.69 ± 0.39
Flavonoids (mg RE/g)	6.48 ± 0.03	6.61 ± 0.03
Anti-nutritional factors		
Glucosinolates (µmol/g)	20.60 ± 0.10	18.80 ± 0.11
Phytic acid (g/100 g)	1.80 ± 0.01	1.24 ± 0.01
Free phosphorus (g/100 g)	0.07 ± 0.001	0.16 ± 0.001
Tannins (mg/g)	5.48 ± 0.03	3.30 ± 0.02
Fatty acid profile (gFAME/100 g Total FAME)		
Caproic, acid C6:0	0.10 ± 0.03	0.02 ± 0.01
Caprylic acid, C8:0	0.13 ± 0.08	0.02 ± 0.01
Capric acid, C10:0	0.08 ± 0.03	0.03 ± 0.01
Myristic acid, C14:0	0.24 ± 0.04	0.12 ± 0.03
Pentadecanoic acid, C15:0	0.19 ± 0.02	0.07 ± 0.05
Pentadecenoic acid, C15:1	0.07 ± 0.03	n.d.
Palmitic acid, C16:0	7.50 ± 1.39	6.85 ± 1.21
Palmitoleic acid, C16:1	0.72 ± 0.23	0.72 ± 0.19
Heptadecanoic acid, C17:0	0.13 ± 0.04	0.12 ± 0.02
Heptadecenoic acid, C17:1	0.18 ± 0.06	0.17 ± 0.08
Stearic acid, C18:0	1.89 ± 0.02	1.45 ± 0.05
Oleic acid, C18:1	57.19 ± 2.91	58.85 ± 2.15
Linoleic acid (LA), C18:2n-6	22.10 ± 1.60	23.73 ± 1.81
Arachidic acid, C20:0	n.d.	0.06 ± 0.02
Gamma-linolenic acid (GLA), C18:3n-6	n.d.	0.06 ± 0.03
Alpha-linolenic acid (ALA), C18:3n-3	8.51 ± 0.39	6.37 ± 0.86
Conjugated linoleic acids (CLAs), C18:(1n7;1n11)	0.30 ± 0.07	0.40 ± 0.05
Octadecatetraenoic acid (EDA), (C18:4n3)	0.52 ± 0.08	0.72 ± 0.03
Eicosadienoic acid, C20:2n6	n.d.	0.04 ± 0.02
Arachidonic acid (ARA), C20:(4n6)	0.15 ± 0.19	n.d.
Other fatty acids	n.d.	0.20 ± 0.01
Fatty acid classification		
Saturated fatty acids (SFA)	10.26 ± 1.39	8.73 ± 0.98
Monounsaturated fatty acids (MUFA)	58.16 ± 2.68	59.75 ± 1.98
Polyunsaturated fatty acids (PUFA)	31.59 ± 1.53	31.32 ± 1.09
Unsaturated fatty acids (UFA)	89.74 ± 1.16	91.07 ± 1.23
Omega-3 fatty acids (Ω3)	9.03 ± 0.06	7.09 ± 0.08
Omega-6 fatty acids (Ω6)	22.25 ± 1.47	23.83 ± 0.98
Omega-6 to omega-3 dietary ratio (Ω6/Ω3)	2.46 ± 0.17	3.36 ± 0.15

Note: Raw rapeseed cakes (RRC); fermented rapeseed cakes (FRC); dry matter (DM); digestibility dry matter (dDM); organic matter (OM); digestibility organic matter (dOM); crude protein (CP); digestibility crude protein (dCP); ether extract (EE); crude fiber (CF); nitrogen-free extractives (NFEs); GAEs (gallic acid equivalents); linoleic acid (LA); gamma-linolenic acid (GLA); alpha-linolenic acid (ALA); conjugated linoleic acids (CLAs); eicosadienoic acid (EDA); arachidonic acid (ARA); saturated fatty acids (SFAs); monounsaturated fatty acids (MUFAs); polyunsaturated fatty acids (PUFAs); unsaturated fatty acids (UFAs); omega-3 fatty acids (Ω3s); omega-6 fatty acids (Ω6s); omega-6-to-omega-3 dietary ratio (Ω6/Ω3); not detected (n.d.) Values represent the mean of 3 analytical determinations performed on representative samples collected from the same production batch of each rapeseed cake product used in the feeding trial.

**Table 2:** Ingredients and chemical composition of the broiler diets (% as fed).

Diet Composition, %	Starter Phase (0–10 Days)	Grower Phase (11–24 Days)			Finisher Phase (>25 Days)		
		SBM	RRC	FRC	SBM	RRC	FRC
Corn	37.29	41.75	36.07	36.07	48.13	42.60	42.60
Wheat	15.00	15.00	15.00	15.00	15.00	15.00	15.00
Soybean meal	38.98	35.20	22.25	22.25	30.00	16.80	16.80
Rapeseed cake	-	-	20.00	20.00	-	20.00	20.00
Vegetable oil	3.70	3.83	2.65	2.65	3.00	1.87	1.87
Calcium carbonate	1.49	1.15	0.94	0.94	1.00	0.82	0.82
Monocalcium phosphate	1.35	1.03	0.92	0.92	0.82	0.71	0.71
Salt	0.42	0.42	0.39	0.39	0.42	0.39	0.39
L—lysine	0.23	0.17	0.36	0.36	0.20	0.40	0.40
DL—methionine	0.37	0.31	0.26	0.26	0.31	0.26	0.26
L—threonine	0.10	0.07	0.10	0.10	0.06	0.09	0.09
Choline	0.05	0.05	0.05	0.05	0.05	0.05	0.05
Phytase	0.01	0.01	0.01	0.01	0.01	0.01	0.01
Vitamin–mineral premix ^1^	1.00	1.00	1.00	1.00	1.00	1.00	1.00
Total ingredients	100	100	100	100	100	100	100
**Dietary nutritional value**							
Metabolizable energy, kcal/kg	2988.22	3050.00	3050.00	3050.00	3100.00	3100.00	3100.00
Crude protein, %	23.00	21.50	21.50	21.50	19.50	19.50	19.50
Crude fat, %	5.74	6.01	7.40	6.48	5.39	6.83	5.90
**Fatty acids profile, g FAMEs/100 g FAMEs**							
Capric acid (C 10:0)	0.04	0.04	0.03	0.03	0.04	0.03	0.02
Myristic acid (C 14:0)	0.23	0.23	0.24	0.21	0.26	0.27	0.23
Palmitic acid (C 16:0)	12.62	12.60	10.65	10.91	12.70	10.56	10.83
Palmitoleic acid (C 16:1)	0.21	0.21	0.41	0.37	0.22	0.44	0.40
Stearic acid (C 18:0)	4.05	4.02	3.11	3.16	3.84	2.90	2.91
Oleic acid (C 18:1)	24.72	24.83	37.79	35.51	25.60	39.48	37.24
Linoleic acid (LA) (C 18:2n-6)	50.98	50.97	39.56	42.56	50.66	38.36	41.47
Alpha-linolenic acid (ALA) (C 18:3n-3)	5.77	5.70	6.61	5.69	5.23	6.33	5.27
Octadecatetraenoic acid (C18:4n-3)	0.12	0.13	0.29	0.32	0.16	0.33	0.37

Note: SBM, control diet; RRC, control diet + 20% raw rapeseed cakes; FRC, control diet + 20% fermented rapeseed cakes. ^1^ Content per kg diet: 1,100,000 IU vitamin A; 200,000 IU vitamin D3; 2700 IU vitamin E; 300 mg vitamin K; 200 mg vitamin B1; 400 mg vitamin B2; 1485 mg pantothenic acid; 2700 mg nicotinic acid; 300 mg vitamin B6; 4 mg vitamin B7; 100 mg vitamin B9; 1.8 mg vitamin B12; 2000 mg vitamin C; 8000 mg manganese; 8000 mg iron; 500 mg copper; 6000 mg zinc; 37 mg cobalt; 152 mg iodine; 18 mg selenium, FAMEs—fatty acid methyl esters; Linoleic acid (LA); Alpha-linolenic acid (ALA).

**Table 3:** Proximate composition, collagen and pH value of thigh and breast chicken meat samples.

Specification	Thigh					Breast				
	SBM	RRC	FRC	SEM	*p*-Value	SBM	RRC	FRC	SEM	*p*-Value
Moisture (%)	75.46 ^ab^	76.72 ^a^	74.13 ^b^	0.541	0.007	77.33 ^a^	77.35 ^a^	75.07 ^b^	0.403	0.0001
Protein (%)	18.98	19.39	19.75	0.501	0.559	21.68	22.09	23.29	0.512	0.082
Collagen (%)	0.93 ^b^	0.89 ^b^	1.08 ^a^	0.033	0.001	0.79 ^b^	0.79 ^b^	0.98 ^a^	0.028	0.0001
pH (value)	6.13	6.02	6.17	0.034	0.542	6.12	6.05	6.07	0.022	0.529

SBM, control diet; RRC, control diet + 20% raw rapeseed cakes; FRC, control diet + 20% fermented rapeseed cakes; 8 samples were analyzed per meat type and experimental group SEM, standard error of the mean. ^a,b^ Mean values within a row having different superscripts are significantly different at *p* ≤ 0.05.

**Table 4:** Effects of raw and fermented rapeseed cakes dietary inclusion on fatty acid composition of broiler meat samples.

Specification	Thigh					Breast				
	SBM	RRC	FRC	SEM	*p*-Value	SBM	RRC	FRC	SEM	*p*-Value
	mg Fatty Acid/100 g Meat					mg Fatty Acid/100 g Meat				
ΣSFA	102.36 ^a^	75.39 ^b^	85.16 ^b^	3.920	0.001	101.73 ^a^	74.77 ^b^	97.52 ^a^	3.65	0.0001
C 10:0	0.12 ^a^	0.08 ^b^	0.12 ^a^	0.013	0.043	0.14	0.13	0.10	0.017	0.297
C 14:0	1.78 ^a^	1.61 ^ab^	1.36 ^b^	0.105	0.035	1.80 ^a^	1.26 ^b^	2.02 ^a^	0.093	0.0001
C 15:0	0.33	0.35	0.44	0.048	0.236	0.32 ^b^	0.72 ^b^	2.28 ^a^	0.169	0.0001
C 16:0	72.59 ^a^	50.16 ^b^	57.87 ^b^	2.790	0.0001	71.52 ^a^	48.11 ^b^	64.69 ^a^	2.800	0.0001
C 17:0	0.78	0.78	0.76	0.062	0.931	0.69	0.65	0.60	0.051	0.493
C 18:0	25.54	21.68	23.26	1.150	0.089	25.99 ^a^	22.87 ^b^	26.81 ^a^	0.747	0.005
C 20:0	0.59	0.63	0.79	0.088	0.239	0.92 ^a^	0.62 ^ab^	0.44 ^b^	0.098	0.011
C24:0	0.42	0.39	0.48	0.095	0.785	0.36	0.42	0.58	0.066	0.093
ΣMUFA	187.20	168.61	185.54	7.420	0.180	183.90 ^ab^	162.40 ^b^	206.47 ^a^	9.03	0.012
C 14:1	0.36 ^a^	0.17 ^b^	0.16 ^b^	0.027	0.0001	0.34 ^a^	0.12 ^b^	0.18 ^b^	0.023	0.0001
C 15:1	1.73 ^a^	1.16 ^ab^	1.06 ^b^	0.162	0.023	1.64 ^a^	1.48 ^a^	0.84 ^b^	0.137	0.002
C 16:1	16.28 ^a^	9.43 ^b^	11.41 ^b^	0.754	0.0001	14.14 ^a^	7.94 ^b^	10.77 ^b^	0.819	0.0001
C 17:1	0.46	0.49	0.34	0.074	0.352	0.58	0.68	0.54	0.039	0.055
C 18:1n9c	166.61	156.04	171.69	6.690	0.271	165.29 ^ab^	150.39 ^b^	193.60 ^a^	8.150	0.006
C 22:1n9	0.17 ^a^	0.09 ^b^	0.05 ^b^	0.015	0.009	0.18	0.26	0.20	0.060	0.670
C 24:1n9	1.60 ^a^	1.23 ^b^	0.85 ^c^	0.079	0.0001	1.75 ^a^	1.52 ^a^	0.44 ^b^	0.184	0.0001
ΣPUFA	131.51	124.91	119.54	6.050	0.397	126.85 ^a^	108.82 ^ab^	96.05 ^b^	7.62	0.037
Σn-6	120.39	109.86	106.41	5.60	0.217	115.24 ^a^	94.32 ^ab^	80.13 ^b^	6.37	0.007
C 18:2n6	109.01	98.91	97.60	4.920	0.232	104.61 ^a^	83.90 ^ab^	78.11 ^b^	5.970	0.017
C 18:3n6	n.d.	0.07	0.04	0.011	0.183	0.08 ^b^	0.07 ^b^	0.24 ^a^	0.008	0.0001
C 20:2n6	1.59	1.47	1.43	0.086	0.399	1.60	1.71	2.04	0.223	0.368
C 20:3n6	1.14 ^a^	0.88 ^b^	0.79 ^b^	0.064	0.004	1.39	1.21	1.05	0.187	0.452
C20:4n6	7.75	7.56	5.85	0.634	0.098	6.64 ^a^	6.46 ^a^	3.09 ^b^	0.734	0.006
C22:2n6	0.36	0.44	0.34	0.065	0.528	0.36 ^ab^	0.39 ^a^	0.23 ^b^	0.041	0.050
C22:3n6	0.31	0.32	0.27	0.059	0.172	0.16	0.31	0.26	0.050	0.101
C22:4n6	0.33	0.20	0.22	0.047	0.115	0.45	0.26	0.29	0.089	0.261
Σn-3	10.83 ^c^	14.78 ^a^	12.92 ^b^	0.502	0.0001	11.30 ^ab^	14.21 ^a^	9.96 ^b^	0.868	0.014
C 18:3n3	7.65 ^b^	10.95 ^a^	10.04 ^a^	0.406	0.0001	7.45 ^ab^	9.37 ^a^	6.91 ^b^	0.542	0.014
CLA (C 18:2)	0.26	0.27	0.21	0.026	0.282	0.31	0.30	0.40	0.058	0.412
C 18:4n3	0.34	0.35	0.39	0.055	0.804	0.50	0.67	0.52	0.059	0.110
C 20:3n3	1.05 ^a^	0.91 ^ab^	0.80 ^b^	0.040	0.003	1.26 ^a^	1.03 ^ab^	0.75 ^b^	0.100	0.009
C 20:5n3	0.34	0.26	0.35	0.081	0.692	0.32	0.33	0.50	0.072	0.180
C22:5n3	0.88	1.02	0.90	0.108	0.071	0.961	1.465	1.116	0.204	0.234
C22:6n3	0.75 ^b^	1.09 ^a^	0.63 ^b^	0.080	0.003	0.82 ^b^	1.34 ^a^	0.86 ^b^	0.151	0.050
n-6/n-3	46.94 ^a^	27.64 ^b^	32.25 ^b^	2.09	0.0001	42.36 ^a^	23.29 ^b^	32.02 ^b^	2.26	0.0001
SFA/UFA	1.36 ^a^	0.95 ^b^	1.09 ^b^	0.052	0.0001	1.36 ^a^	0.96 ^b^	1.30 ^a^	0.048	0.0001
PUFA/MUFA	2.97	2.75	2.52	0.140	0.115	2.86 ^a^	2.34 ^ab^	1.88 ^b^	0.175	0.005
PUFA/SFA	1.29 ^b^	1.65 ^a^	1.41 ^b^	0.047	0.0001	1.25 ^a^	1.45 ^a^	0.99 ^b^	0.070	0.001

SBM, control diet; RRC, control diet + 20% raw rapeseed cakes; FRC, control diet + 20% fermented rapeseed cakes; 8 samples were analyzed per meat type and experimental group; SEM, standard error of the mean. ^a–c^ Mean values within a row having different superscripts are significantly different at *p* ≤ 0.05. Abbreviations: ΣSFA, sum of saturated fatty acids; ∑MUFA, sum of monounsaturated fatty acids; ΣPUFA, sum of polyunsaturated fatty acids; n-6/n-3, ratio of omega-6 fatty acids/omega-3 fatty acids; SFA/UFA, saturated fatty acid/unsaturated fatty acid; PUFA/MUFA, polyunsaturated fatty acid/monounsaturated fatty acid; PUFA/SFA, polyunsaturated fatty acid/saturated fatty acid; not detected (n.d.).

**Table 5:** Effects of raw and fermented rapeseed cakes dietary inclusion on health indices.

Specification	Thigh					Brest				
	SBM	RRC	FRC	SEM	*p*-Value	SBM	RRC	FRC	SEM	*p*-Value
Quality indices										
LA/ALA	14.24 ^a^	9.04 ^b^	9.77 ^b^	0.299	0.0001	14.03 ^a^	8.97 ^c^	11.38 ^b^	0.351	0.0001
EPA + DHA	1.09	1.35	0.98	0.106	0.072	1.14	1.66	1.35	0.155	0.080
UI	483.35	456.58	455.23	21.00	0.576	470.21	417.56	423.53	25.5	0.307
Nutritional indices										
PI	182.29	181.62	165.46	9.18	0.366	176.66	165.41	135.83	12.3	0.084
NVI	2.65 ^b^	3.54 ^a^	3.37 ^a^	0.054	0.0001	2.68 ^b^	3.60 ^a^	3.41 ^a^	0.062	0.0001
AI	0.25 ^a^	0.19 ^c^	0.21 ^b^	0.005	0.0001	0.25 ^a^	0.19 ^b^	0.24 ^a^	0.007	0.0001
TI	0.54 ^a^	0.40 ^c^	0.45 ^b^	0.011	0.0001	0.54 ^a^	0.43 ^b^	0.54 ^a^	0.015	0.0001
HH ratio	4.02 ^c^	5.45 ^a^	4.91 ^b^	0.114	0.0001	3.99 ^b^	5.24 ^a^	4.34 ^b^	0.155	0.0001
HPI	217.67 ^a^	150.25 ^b^	180.10 ^ab^	15.6	0.026	213.30 ^a^	134.78 ^b^	217.75 ^a^	15.7	0.003
Metabolic indices										
Elongase	35.24 ^b^	43.23 ^a^	40.29 ^a^	1.15	0.001	36.57 ^c^	47.68 ^a^	41.59 ^b^	1.12	0.0001
Thioesterase	4147.45	3711.85	3642.25	178.00	0.129	4000.60 ^a^	3823.46 ^a^	3219.21 ^b^	80.9	0.0001
D9-desaturase	86.72 ^b^	87.79 ^ab^	88.06 ^a^	0.297	0.015	86.36 ^b^	86.73 ^ab^	87.80 ^a^	0.314	0.015
D9-desaturase (C16:1 + C18:1)	65.10 ^b^	69.71 ^a^	69.30 ^a^	0.374	0.0001	64.75 ^b^	68.96 ^a^	69.04 ^a^	0.405	0.0001
D5-desaturase + D6-desaturase	0.09 ^ab^	0.10 ^a^	0.08 ^b^	0.004	0.024	0.09 ^ab^	0.11 ^a^	0.08 ^b^	0.007	0.034
Activity Index	18.48 ^b^	25.73 ^a^	22.96 ^a^	0.891	0.0001	18.75 ^ab^	23.58 ^a^	15.21 ^b^	1.53	0.005

SBM, control diet; RRC, control diet + 20% raw rapeseed cakes; FRC, control diet + 20% fermented rapeseed cakes; 8 samples were analyzed per meat type and experimental group; SEM, standard error of the mean. ^a–c^ Mean values within a row having different superscripts are significantly different at *p* ≤ 0.05. Abbreviations: LA/ALA = (C18:2n-6)/(C18:3n-3); EPA + DHA = (C20:5n-3) + (C22:6n-3); UI = (% monoenoic) + (2 × % dienoic) + (3 × % trienoic) + (4 × % tetraenoic) + (5 × % pentaenoic) + (6 × % hexaenoic); PI = (monoenoic acid × 0.025) + (dienoic acid × 1) + (trienoic acid × 2) + (tetraenoic acid × 4) + (pentaenoic acid × 6) + (hexaenoic acid × 8); NVI = (C18:0 + C18:1n9)/(C16:0); AI = [C12:0 + (4 × C14:0) + C16:0]/(∑UFA); TI = (C14:0 + C16:0 + C18:0)/[(0.5 × MUFA) + (0.5 × ∑n-6 PUFA) + (3 × ∑n-3 PUFA) + (∑n-3 PUFA/∑n-6 PUFA)]; HH ratio = (C18:1 + ∑PUFA)/(C12:0 + C14:0 + C16:0); HPI = (∑UFA)/[C12:0 + (4 × C14:0) + C16:0]; Elongase = (C18:0/C16:0) × 100; Thioesterase = (C16:0/C14:0) × 100; D9-desaturase (C:18:1) = (C18:1n-9/C18:0 + C18:1n-9) × 100; D9-desaturase (C16:1 + C:18:1) = (C16:1n-7 + C18:1n-9/C16:0 + C18:0 + C16:1n-7 + C18:1n-9) × 100; D5-desaturase + D6-desaturase = (C20:2n-6 + C20:4n-6 + C20:5n-3 + C22:5n-3 + C22:6n-3)/(C18:2n-6 + C18:3n-3 + C20:2n-6 + C20:4n-6 + C20:5n-3 + C22:5n-3 + C22:6n-3); activity index = (∑n-3 PUFA)/(C18:3n-3); linoleic acid/α-linolenic acid ratio (LA/ALA); eicosapentaenoic acid + docosahexaenoic acid (EPA + DHA); unsaturation index (UI); peroxidability index (PI); nutritional value index (NVI); atherogenicity index (AI); thrombogenicity index (TI); ratio, hypocholesterolemic/hypercholesterolemic fatty acid ratio (HH ratio); health-promoting index (HPI).

**Table 6:** Effects of raw and fermented rapeseed cakes dietary inclusion on meat texture parameters of broiler meat quality.

Specification	Thigh					Breast				
	SBM	RRC	FRC	SEM	*p*-Value	SBM	RRC	FRC	SEM	*p*-Value
Hardness (g)	609.75	487.50	529.61	59.2	0.431	3197.50	4440.83	4123.23	378	0.060
Adhesiveness (mj)	0.35	0.36	0.29	0.134	0.850	0.33	0.37	0.36	0.065	0.896
Resilience	0.18	0.19	0.16	0.021	0.484	0.23	0.24	0.23	0.0098	0.588
Cohesiveness	0.44	0.45	0.41	0.055	0.838	0.41 ^a^	0.39 ^a^	0.33^b^	0.013	0.0001
Springiness (mm)	2.97	3.42	2.65	0.319	0.292	3.02	2.92	3.28	0.200	0.438
Gumminess (g)	301.30	237.36	262.53	45.9	0.669	1425.96	1932.25	1548.96	154	0.059
Chewiness (mj)	5.22	5.96	5.74	1.13	0.930	42.51	57.36	48.34	5.75	0.191

SBM, control diet; RRC, control diet + 20% unfermented rapeseed cakes; FRC, control diet + 20% fermented rapeseed cakes; 8 samples were analyzed per meat type and experimental group; SEM, standard error of the mean. ^a,b^ Mean values within a row having different superscripts are significantly different at *p* ≤ 0.05.

**Table 7:** Effects of raw and fermented rapeseed cakes dietary inclusion on color-related biochemical parameters of broiler meat during different storage periods (0, 7, and 14 days).

Item	Thigh		Breast	
	MB (mg/g Meat)	MetMb (%)	MB (mg/g Meat)	MetMb (%)
day 0				
SBM	0.63 ^ab^	59.92	0.54	58.66 ^ab^
RRC	0.66 ^a^	58.87	0.57	57.66 ^b^
FRC	0.65 ^ab^	60.14	0.56	59.01 ^ab^
day 7				
SBM	0.61 ^ab^	61.23	0.52	59.88 ^ab^
RRC	0.64 ^ab^	59.90	0.55	58.71 ^ab^
FRC	0.63 ^ab^	60.49	0.54	59.29 ^ab^
day 14				
SBM	0.59 ^b^	60.76	0.51	60.76 ^a^
RRC	0.61 ^ab^	59.44	0.53	59.44 ^ab^
FRC	0.61 ^ab^	59.96	0.52	59.96 ^ab^
Main effect				
Period				
0 d	0.65 ^a^	59.64	0.56 ^a^	58.45 ^b^
7 d	0.63 ^ab^	60.54	0.54 ^ab^	59.30 ^ab^
14 d	0.60 ^b^	60.05	0.52 ^b^	60.05 ^a^
SEM period	0.009	0.339	0.008	0.338
Group				
SBM	0.61	60.64 ^a^	0.52	59.77 ^a^
RRC	0.64	59.40 ^b^	0.55	58.61 ^b^
FRC	0.63	60.19 ^ab^	0.54	59.42 ^ab^
SEM group	0.009	0.339	0.008	0.338
*p*-value				
period	0.002	0.185	0.009	0.006
group	0.072	0.042	0.066	0.054
period × group	0.998	0.904	1.000	0.874

SBM, control diet; RRC, control diet + 20% raw rapeseed cakes; FRC, control diet + 20% fermented rapeseed cakes 8 samples/meat type/group with 3 repeated measures per sample); SEM, standard error of the mean. ^a,b^ Mean values within a row having different superscripts are significantly different at *p* ≤ 0.05. Abbreviations: MB, myoglobin; MetMb, metmyoglobin.

**Table 8:** Effects of fermented and raw rapeseed cakes dietary inclusion on color chicken meat parameters during different storage periods (0, 7, and 14 days).

Color Parameter	Thigh			Breast		
	L*	a*	b*	L*	a*	b*
day 0						
SBM	42.54	−0.03	5.35 ^ab^	43.17 ^ab^	−1.87 ^ab^	2.65 ^bc^
RRC	41.94	0.26	5.92 ^ab^	43.48 ^ab^	−2.09 ^ab^	2.85 ^bc^
FRC	41.22	1.03	4.88 ^b^	41.36 ^ab^	−1.59 ^a^	2.43 ^c^
day 7						
SBM	42.17	0.15	6.09 ^ab^	43.17 ^ab^	−2.19 ^b^	3.67 ^a^
RRC	42.51	0.98	6.32 ^ab^	44.72 ^a^	−2.02 ^ab^	2.89 ^bc^
FRC	39.49	1.09	4.99 ^ab^	41.02 ^b^	−2.01 ^ab^	3.06 ^abc^
day 14						
SBM	43.14	0.65	6.56 ^a^	42.91 ^ab^	−1.92 ^ab^	3.02 ^abc^
RRC	42.61	0.38	5.98 ^ab^	43.53 ^ab^	−2.30 ^b^	3.30 ^ab^
FRC	42.17	0.24	5.33 ^ab^	40.81 ^b^	−1.58 ^a^	3.24 ^ab^
Main effect						
Period						
0 d	41.90	0.42	5.38	42.67	−1.85	2.64 ^b^
7 d	41.39	0.74	5.80	42.97	−2.07	3.21 ^a^
14 d	42.64	0.42	5.96	42.42	−1.93	3.19 ^a^
SEM period	0.493	0.146	0.208	0.450	0.073	0.089
Group						
SBM	42.62 ^a^	0.26 ^b^	6.00 ^a^	43.08 ^a^	−1.99 ^b^	3.11
RRC	42.35 ^ab^	0.54 ^ab^	6.07 ^a^	43.91 ^a^	−2.14 ^b^	3.01
FRC	40.96 ^b^	0.79 ^b^	5.07 ^b^	41.06 ^b^	−1.73 ^a^	2.91
SEM group	0.493	0.146	0.208	0.450	0.073	0.089
*p*-Value						
period	0.200	0.211	0.138	0.686	0.096	0.0001
group	0.041	0.039	0.001	0.0001	0.0001	0.278
period × group	0.582	0.019	0.532	0.882	0.066	0.002

SBM, control diet; RRC, control diet + 20% raw rapeseed cakes; FRC, control diet + 20% fermented rapeseed cakes; 8 samples/meat type/group with 3 repeated measures per sample); SEM, standard error of the mean. ^a–c^ Mean values within a row having different superscripts are significantly different at *p* ≤ 0.05. L* indicates lightness (0 = black, 100 = white); a* represents the red–green component (positive values = red, negative values = green); and b* represents the yellow–blue component (positive values = yellow, negative values = blue).

**Table 9:** Effects of fermented and raw rapeseed cakes dietary inclusion on meat sensory evaluation during different storage periods (0, 7, and 14 days).

Characteristics	Thigh				Breast			
	Odor	Firmness	Consistency	Elasticity	Odor	Firmness	Consistency	Elasticity
day 0								
SBM	4.00 ^b^	4.83 ^a^	4.50 ^ab^	4.83 ^a^	4.25 ^ab^	4.50 ^ab^	4.67 ^a^	4.67 ^a^
RRC	4.08 ^b^	4.67 ^ab^	4.67 ^a^	4.83 ^a^	4.33 ^b^	4.50 ^ab^	4.67 ^a^	4.83 ^a^
FRC	4.25 ^a^	5.00 ^a^	4.50 ^ab^	4.83 ^a^	4.50 ^a^	4.83 ^a^	4.50 ^ab^	4.67 ^a^
day 7								
SBM	2.83 ^c^	3.33 ^cd^	3.33 ^cd^	3.50 ^a^	3.00 ^c^	3.17 ^cd^	3.50 ^bcd^	3.50 ^a^
RRC	3.42 ^abc^	3.83 ^bc^	3.67 ^bc^	4.17 ^a^	3.50 ^bc^	3.67 ^bc^	3.83 ^abc^	4.33 ^a^
FRC	3.92 ^ab^	4.67 ^ab^	4.00 ^abc^	3.67 ^a^	4.08 ^ab^	4.50 ^ab^	4.17 ^ab^	3.67 ^a^
day 14								
SBM	1.92 ^d^	2.00 ^e^	1.83 ^e^	1.50 ^b^	1.92 ^d^	2.17 ^d^	2.17 ^e^	1.50 ^b^
RRC	3.17 ^bc^	2.83 ^de^	2.50 ^de^	1.67 ^b^	2.83 ^c^	3.00 ^cd^	2.83 ^cde^	1.67 ^b^
FRC	3.50 ^abc^	2.50 ^de^	2.17 ^e^	1.67 ^b^	2.83 ^c^	3.00 ^cd^	2.50 ^de^	1.67 ^b^
Main effect								
Period								
0 d	4.11 ^a^	4.83 ^a^	4.56 ^a^	4.83 ^a^	4.36 ^a^	4.61 ^a^	4.61 ^a^	4.72 ^a^
7 d	3.39 ^b^	3.94 ^b^	3.67 ^b^	3.78 ^b^	3.53 ^b^	3.78 ^b^	3.83 ^b^	3.83 ^b^
14 d	2.86 ^c^	2.44 ^c^	2.17 ^c^	1.61 ^c^	2.53 ^c^	2.72 ^c^	2.50 ^c^	1.61 ^c^
SEM period	0.111	0.122	0.123	0.180	0.106	0.133	0.143	0.179
Group								
SBM	2.92 ^b^	3.39 ^b^	3.22	3.28	3.06 ^b^	3.28 ^b^	3.44	3.22
RRC	3.56 ^a^	3.78 ^ab^	3.61	3.56	3.81 ^a^	3.72 ^ab^	3.78	3.61
FRC	3.89 ^a^	4.06 ^a^	3.56	3.39	3.56 ^a^	4.11 ^a^	3.72	3.33
SEM group	0.111	0.122	0.123	0.180	0.106	0.133	0.143	0.179
*p*-value								
period	0.0001	0.0001	0.0001	0.0001	0.0001	0.0001	0.0001	0.0001
group	0.0001	0.001	0.063	0.552	0.0001	0.0001	0.220	0.294
period × group	0.008	0.012	0.370	0.833	0.068	0.129	0.363	0.747

SBM, control diet; RRC, control diet + 20% raw rapeseed cakes; FRC, control diet + 20% fermented rapeseed cakes; *n* = number of samples per group (8 samples/group with 3 repeated measures per sample); SEM, standard error of the mean. ^a–e^ Mean values within a row having different superscripts are significantly different at *p* ≤ 0.05. Attributes of odor intensity and texture intensity: 0 = absent/not perceived, 1 = very weak, 2 = weak, 3 = moderate, 4 = intense, and 5 = very intense.

## Data Availability

The original contributions presented in this study are included in the article Further inquiries can be directed to the corresponding authors.
